# The Effects of Milk and Posterior Intestinal Microorganisms on the Lactation Performance of Dual-Purpose Cattle (*Bos taurus*) Revealed by 16S rRNA Sequencing

**DOI:** 10.3390/microorganisms13020448

**Published:** 2025-02-18

**Authors:** Weitao Wang, Shengchao Ma, Dan Wang, Lei Xu, Menghua Zhang, Mengjie Yan, Kailun Ma, Zexu Hu, Yanyan Shang, Jiangjiang Wei, Xixia Huang

**Affiliations:** College of Animal Science, Xinjiang Agricultural University, Urumqi 830052, China

**Keywords:** dual-purpose cattle, milk and hindgut, 16S rRNA sequencing, microbial community diversity, endogenous and exogenous invasion, correlation, effect on lactation performance

## Abstract

The aim of this research was to employ 16S rRNA high-throughput sequencing to thoroughly explore the interplay between milk and hindgut microbial communities and the effects of microorganisms in milk and the hindgut on the dairy quality of XJBC and CSC. In this study, 96 XJBC milk samples, 94 XJBC hindgut samples, 100 CSC milk samples, and 93 CSC hindgut samples were collected for microbial community analysis. The 16S rRNA sequencing data revealed that the microbial species richness in the milk of CSC exceeded that of XJBC, whereas the opposite was true for the hindgut microbial communities. A chi-square test was conducted using SPSS 19.0. The milk and posterior intestinal microbiota between individuals were analyzed with the Pearson chi-square test, maximum likelihood ratio, and Fisher’s exact test. Nongenetic factors substantially influenced microbial community dynamics in both milk and the hindgut. In the milk of dairy cows, a significant negative correlation was observed between one genus and milk protein production. Nine genera were significantly negatively correlated with milk fat production, whereas one genus was positively correlated. Additionally, six genera were negatively correlated with lactose production, and two genera exhibited positive correlations. Notably, *Phascolarctobacterium* and *Turicibacter* were identified as genera originating from the hindgut, which led to reduced milk quality. In the hindgut microbial community of dairy cows, seven genera were significantly negatively associated with milk fat production, whereas one genus was positively associated with milk fat production. These findings indicate that certain mammary microorganisms may migrate from the hindgut, either endogenously or exogenously, disrupting the equilibrium of the mammary microbial community in dairy cows and potentially leading to inflammation. By enhancing feeding conditions and standardizing production practices, the invasion of harmful flora into mammary tissues can be minimized, reducing the risk of inflammation and thereby preserving the health of dairy cows and enhancing milk quality.

## 1. Background

Cow’s milk is a nutrient-dense food that is rich in minerals, milk fat, milk protein, and lactose [1]. As an important source of daily nutrition, its production and quality have received increasing attention. Traditionally, milk has been perceived as a “clean” and sterile product, yet it actually harbors a diverse microbial community comprising both beneficial and harmful microorganisms [2,3]. These microorganisms not only influence milk quality but can also lead to mastitis in dairy cows. Chronic mastitis, referred to as “the cancer that will not die” due to its treatment challenges and high recurrence rate, significantly increases the culling rate of lactating cows, impacts farm economic income, and diminishes animal welfare. Initially, mastitis was attributed to Streptococcus lactis and Staphylococcus aureus; however, subsequent research has revealed a broader spectrum of bacterial species, including fungi, Mycoplasma, and algae [4]. In addition to Escherichia coli and Staphylococcus aureus, Klebsiella, coagulase-negative Staphylococcus (CNS), and Proteus are also implicated in mastitis development [5,6]. Research has further revealed correlations between milk microorganisms and milk composition, with Bifidobacterium and lactic acid bacteria capable of altering milk sugar, protein, and fat contents, thereby impacting the nutritional value of dairy products [7,8]. Consequently, the microbial composition of milk has emerged as a focal point of interest.

The origins of milk microorganisms are multifaceted, with some derived from genetic inheritance and others from external and internal environments [9,10]. Previous studies have indicated that 32 microorganisms exhibit heritability [11]. Environmental flora, such as those from the Enterobacteriaceae, Pseudomonadaceae, Streptococcaceae, Xanthomonadaceae, Moraxellaceae, and Ruminococcaceae families, can enter mammary glands from both in vitro and in vivo sources (e.g., the digestive tract), leading to a decline in milk quality and an increased risk of mastitis in dairy cows [2,12]. Genetically sourced genera can be optimized through selective breeding.

During the milk production process, environmental microorganisms can penetrate the mammary gland through compromised mammary tissue and unsealed teats in cows, thereby disrupting the equilibrium of the mammary microbiota [13]. Furthermore, the method of calving and endogenous invasion markedly affect the microbial diversity in milk, with vaginal calving resulting in significantly richer biodiversity than cesarean delivery does [14,15,16]. To increase production efficiency, contemporary dairy cattle rearing is predominantly intensive [17]. Under these intensive conditions, cows often have prolonged contact with their own hindgut excreta, leading to the entry of microorganisms into the mammary gland through damaged tissues and teats, which in turn alters the microbial diversity of milk [13]. This is particularly crucial for studying microorganisms that, despite constituting a minor fraction of the microbial community, still play a significant role [18]. By mitigating the impact of exogenous microorganisms on the mammary bacterial balance, mastitis caused by disruptions in the mammary flora can be prevented. This can be achieved by promptly cleaning the feeding environment, rationalizing the feed preparation, and standardizing milking operations to minimize damage to the teats.

Furthermore, the gut microbiota plays an essential role in the health of dairy cows. The hindgut microflora is both diverse and abundant, contributing to the digestion and absorption of nutrients within the gastrointestinal tract, eliciting specific immune responses, and protecting against pathogen colonization [19,20]. Simultaneously, dysbiosis within the gut microbiota can precipitate systemic inflammatory diseases, intestinal disorders, liver diseases, hypertension, and immune system dysfunctions in the host [21]. Many studies have shown that ecological imbalances in the gut microbiota, often linked to high-concentrate diets, result in shifts in the gut microbiota composition and bacterial overgrowth [22]. XJBC and CSC are the predominant dual-purpose breeds in the Xinjiang region; they are valued by farmers, herders, and enterprises for their excellent characteristics and advantages and have become a cornerstone of Xinjiang’s livestock industry [23]. Changes in the microbial flora of the mammary gland can lead to decreases in milk production and quality [24].

Given the significant impact of milk and hindgut microbiota on dairy cow health and milk quality, this study hypothesizes that the composition and diversity of milk and hindgut microbiota are closely linked to lactation performance in dual-purpose cattle. Specifically, we propose that lactation stage, age at first calving, and parity influence the milk and hindgut microbiota of Xinjiang brown cattle (XJBC) and Chinese Simmental cattle (CSC); milk microbiota directly modulates lactation performance, while hindgut microbiota may exert indirect effects via interactions with milk microbial communities, nutrient absorption, and systemic health; and identifying key microbial taxa associated with lactation outcomes could inform strategies to enhance milk production and quality.

This study aims to characterize the milk and hindgut microbiota of XJBC and CSC through high-throughput sequencing of the 16S rRNA V3–V4 regions; analyze the effects of lactation stage, age at first calving, and parity on microbiota composition; evaluate the relationship between milk microbiota and lactation performance; explore the ecological connections between hindgut and milk microbiota; and establish foundational data for optimizing cattle feeding strategies.

**Figure 1:**
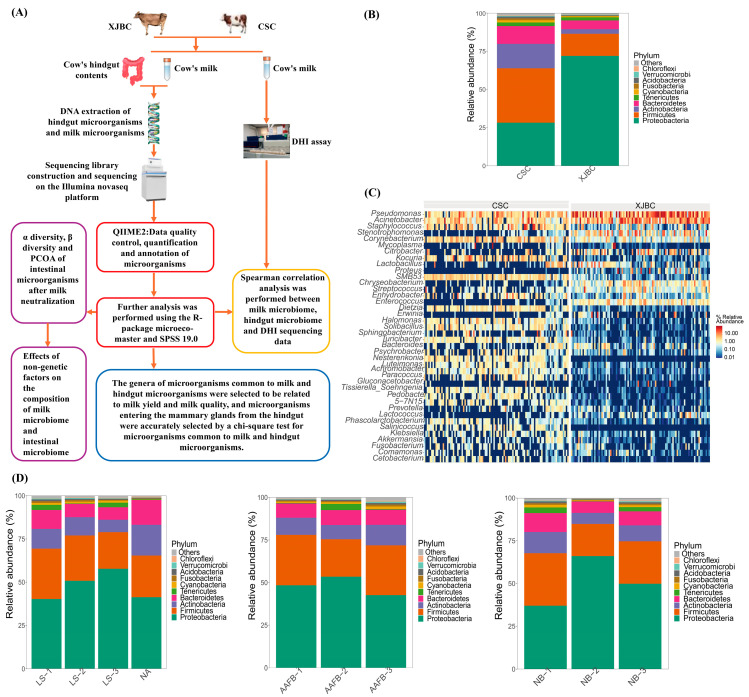
Differences in microbial and nongenetic factors in XJBC and CSC milk: (**A**) Workflow of this study. (**B**) Microbial diversity at the XJBC and CSC phylum levels. (**C**) Microbial diversity at the XJBC and CSC genus levels. (**D**) Effects of nongenetic factors on microbes in XJBC and CSC milk: LS: indicates different lactation stages; AAFB: indicates different first calving months of age; NB: indicates different litter sizes.

In this investigation (Figure 1A), we collected 383 milk and hindgut content samples from XJBC and CSC, followed by sequencing the 16S rRNA V3–V4 regions using the Illumina NovaSeq platform. This approach enabled the taxonomic identification of bacterial genera/species and the quantification of their relative abundances. Ultimately, we identified the microbial communities that upregulate and downregulate lactation performance, revealed the impact of hindgut microbiota on milk microbiota, and provided foundational data for feeding strategies to optimize the health and milk quality of these cattle breeds, thereby supporting the improvement of genetic traits in dairy cows.

## 2. Materials and Methods

### 2.1. Animals

In this study, 100 CSC and 100 XJBC were selected from Chuangjin Agricultural and Animal Husbandry Co., Ltd. (Yili City, China) and Xinhe Breeding Cattle Farm Breeding Co., Ltd. (Yili City, China), respectively. The complete genealogies and breeding records of these 100 CSC and 100 XJBC are available, and both breeds received TMR premix-based feed during lactation.

### 2.2. Sample Collection

First, we collected milk and hindgut content samples from 100 XJBC and 100 CSC, and some samples of substandard quality were eliminated. A total of 187 milk samples (XJBC, *n* = 94; CSC, *n* = 93) and 197 (XJBC, *n* = 96; CSC, *n* = 98) hindgut content samples were ultimately retained. During the sampling period, weather with less fluctuations in temperature and humidity was selected, disposable sterile long-arm gloves were used, and the contents of the hindgut of each cow were collected, preserved on dry ice with two sterile tubes, and then transferred to an ultralow-temperature freezer at −80 °C. Sterile gloves were used to collect milk samples; the teats were first sterilized with alcohol prior to milking, the milk samples were preserved in two sterile tubes, the samples used for the analysis of the composition of the milk were supplemented with potassium dichromate and stored at 4 °C, and then the samples were sent to the Xinjiang Uygur Autonomous Region Dairy Management Office for DHI determination using a Milkoscan FT++Fossomatie 5000 multifunctional analyzer from Hillerod, Denmark. The remaining milk samples for 16S rRNA sequencing were temporarily stored in liquid nitrogen and transferred to the laboratory, where they were frozen at −80 °C.

### 2.3. DNA Extraction and 16S rRNA Sequencing

Total genomic DNA was extracted from samples using a TGuide S96 Magnetic Soil/Stool DNA Kit (Tiangen Biotech Co., Ltd., Beijing, China) according to the manufacturer’s instructions. After quality control of the DNA samples, the hypervariable region V3–V4 of the bacterial 16S rRNA gene was amplified with the primer pairs 338F: 5′-ACTCCTACGGGAGGCAGCA-3′ and 806R: 5′-GGACTACHVGGGTWTCTAAT-3′. The PCR products were subjected to agarose gel electrophoresis and purified with an Omega DNA purification kit (Omega Inc., Norcross, GA, USA). The purified PCR products were collected, and paired-end sequencing (2 × 250 bp) was performed on the Illumina NovaSeq 6000 platform (Illumina, Inc., San Diego, CA, USA).

### 2.4. 16S rRNA Sequencing Data Processing and Bioinformatics Analysis

A total of 68,897,197 forward and reverse sequences of 384 samples were obtained from the 16S rRNA amplicon sequencing data in this study. The tags and primer sequences in the original sequences were removed using QIIME2 (version 2023.9.0) software [25], and then the sequencing sequences were quality controlled, denoised (denoise), and spliced, and chimeras were removed using the built-in plug-in DADA2 of QIIME2 software, resulting in a total of 56,844,602 high-quality sequences, which were obtained and used in the subsequent analysis. The operational taxonomic units (OTUs) and the abundance table of the OTUs were obtained by 100% similarity clustering. The QIIME2 software’s classify-sklearn algorithm and the Greengenes (2022.10 Greengenes2) database were used, and the reference sequences were cut according to the region (V3–V4) of the 16S rRNA gene used in this study, both using default parameters and a pretrained NaveBayes classifier for species annotation performed on the feature sequences of each OTU to obtain species classification information.

The following analysis was performed using microeco-master software (v1.10.0). First, the influence of nongenetic factors on the abundance of milk or postgut microbes was analyzed by α and β diversity. To analyze the effects of nongenetic factors on microbial diversity in milk/postintestinal contents, sequencing samples were divided by the controlled variable method according to the following levels. First calving months of age: 20–27 months of age was the first level, 28–30 was the second level, and >30 was the third level. Cow parity was classified as follows: first-level pregnancy, second-level pregnancy, and third-level pregnancy and above. Lactation stage was divided into three stages as follows: early lactation, from the beginning of calving to the end of the tenth week; mid-lactation, from the eleventh week to the end of the twentieth week after giving birth; and late lactation, from the twenty-first week after giving birth to dry milk. The sample sizes at the different levels are shown in Table 1. The species diversity within individual samples was investigated by α diversity analysis, and the statistics of each sample’s Chao1 index and the sample rank abundance curves were plotted; the similarities and differences among microbial communities were further explored by β diversity analysis, and the Bray–Curtis distance was calculated from the distance matrix and the Bray–Curtis index. Sample hierarchical clustering (UPGMA) trees, NMDS analysis, sample clustering heatmaps and sample PCA and principal coordinate analysis (PCoA) plots (with grouping information), and box-and-line plots based on a variety of distances were constructed based on the distance matrices. At the level of the taxonomic composition of the species, the compositional difference in species abundance among different samples (groups) was further measured through the analysis of the significance of differences between the groups, and the differences in species abundance were searched for among the different groups with significantly different biomarkers. Based on the compositional distribution of species in each sample, association networks were constructed, and network analysis, correlation heatmap, RDA/CCA analysis, and environmental factor sequencing regression analysis were performed. Based on the 16S rRNA or ITS gene sequencing results, through functional prediction analysis, gene functions or phenotypes of the samples were predicted, and the functional genes and phenotypic abundance were calculated. Finally, LEfSe analysis was performed to identify microbial species that differed significantly in abundance.

### 2.5. Correlation Analysis and Chi-Square Test

Correlation analysis between microbial abundance in milk or the hindgut, SCC, milk fat percentage, milk protein percentage, and lactose percentage was performed using microeco-master software (version 1.10.0). Chi-square tests (Pearson chi-square test, Fisher exact test and maximum likelihood method) were used to analyze the associations between milk microbes and hindgut microbes. The chi-square tests were performed in SPSS 19.0. We used all 16S RNA-sequenced samples for correlation analyses and the chi-square test. In the statistical analysis, *p* < 0.05 was considered significant, and *p* < 0.01 was considered extremely significant. In the statistical analysis, *p* < 0.05 was considered significant, and *p* < 0.01 was considered extremely significant. A value of *p* < 0.05 corresponds to one asterisk ‘*’, *p* < 0.01 corresponds to two asterisks ‘**’, and *p* < 0.001 corresponds to three asterisks ‘***’.

## 3. Results

### 3.1. Microbial Composition of Milk from XJBC and CSC

As shown in Figure 1, relative abundance histograms demonstrate the composition of the top 10 species with the highest abundance values at the phylum level for each sample. The analysis revealed significant differences in the bacterial community structure at the phylum level between XJBC and CSC milk samples. Among all the samples, five phyla had relative abundance values greater than 1%, namely, Proteobacteria, Firmicutes, Bacteroidetes, Actinobacteria, and Tenericutes, which together accounted for 85% of the total abundance. Notably, the highest abundance values of Firmicutes were found in milk samples from CSC, whereas the highest abundance values of Proteobacteria were observed in milk samples from XJBC.

As illustrated in Figure 1A, a relative abundance heatmap was generated to display the composition of the 40 microbial species with the highest abundance values in each sample at the genus level. A heatmap is an intuitive visualization tool that indicates the magnitude of a value through color changes, with a gradient from blue to red representing an increase in the relative abundance of a microorganism. The experimental results revealed a significant difference in the community structure of the bacterial flora in the XJBC and CSC milk samples at the genus level. Specifically, the relative abundances of microorganisms such as Pseudomonas, Acinetobacter, Stenotrophomonas, Citrobacter, Corynebacterium, Enterococcus, and Lactobacillus were greater in the milk of XJBC. In contrast, the relative abundances of Pseudomonas, Lactobacillus, and Corynebacterium were greater in the milk of CSC. Additionally, the relative abundances of these three genera—Pseudomonas, Lactobacillus, and Corynebacterium—were elevated in both breeds, suggesting that they play important roles in the microbial communities of both types of cow’s milk.

The experiment was divided into three stages based on the age at first calving of XJBC and CSC. The distribution of the bacterial community structure was relatively stable across these stages. In the first stage, Firmicutes and Actinobacteria had the highest abundance values; in the second stage, Proteobacteria dominated; and in the third stage, Bacteroidetes had the highest abundance values. Similarly, cows at different lactation stages were categorized into three levels, revealing a stable distribution of the bacterial community structure. In the first lactation stage, Firmicutes, Actinobacteria, and Tenericutes had the highest abundance values, whereas Proteobacteria dominated in the third lactation stage. Furthermore, cattle were divided into three levels according to parity, and the bacterial community structure remained relatively stable. At the first parity level, Firmicutes, Actinobacteria, and Bacteroidetes had the highest abundance values, whereas Proteobacteria were predominant at the second parity level.

### 3.2. Microbial α Diversity in the Milk of XJBC and CSC

**Figure 2:**
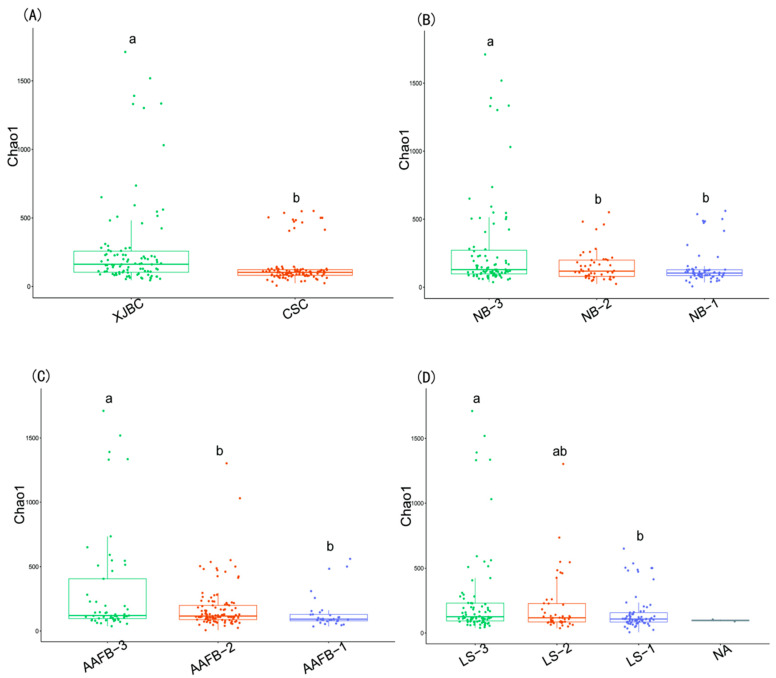
α Diversity estimates of microbial communities in the milk of dairy cows. (**A**–**D**), respectively: (**A**) indicates different breeds; (**B**) indicates different parity of cows; (**C**) indicates different first calving months of age; (**D**) indicates different stages of lactation. Different lowercase letters indicate significant differences (*p* < 0.05), and the same lowercase letters indicate nonsignificant differences (*p* > 0.05).

As shown in Figure 2, the α-Chao1 index was represented using a box plot. The results of the Chao1 index indicated that the microbial diversity in the milk of XJBC was generally greater than that in the milk of CSC, and this difference between breeds was significant (*p* < 0.05). By categorizing XJBC and CSC based on different ages at first calving into three levels, we found that the distribution of the bacterial community structure was relatively stable. Specifically, the third level presented significantly greater abundance values than the first and second levels (*p* < 0.05).

Similarly, when cows were divided into three levels according to different stages of lactation, a stable distribution of the bacterial community structure was observed. The α-Chao1 index was significantly greater in the third lactation stage than in the first stage (*p* < 0.05). Additionally, when cows were categorized into three levels based on parity, the bacterial community structure remained stable, and the α-Chao1 index was significantly greater at the third parity level than at the first and second levels (*p* < 0.05).

### 3.3. Results of PCoA and β Diversity in the Milk of XJBC and CSC

**Figure 3:**
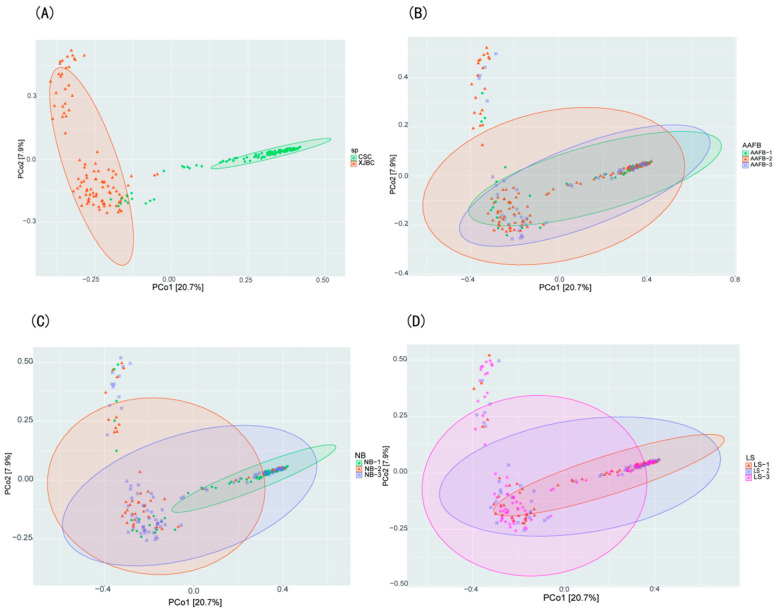
PCOA maps of milk microbes in dairy cows: (**A**) indicates XJBC versus CSC; (**B**) indicates different first calving months of age; (**C**) indicates different parities; (**D**) indicates different stages of lactation.

**Figure 4:**
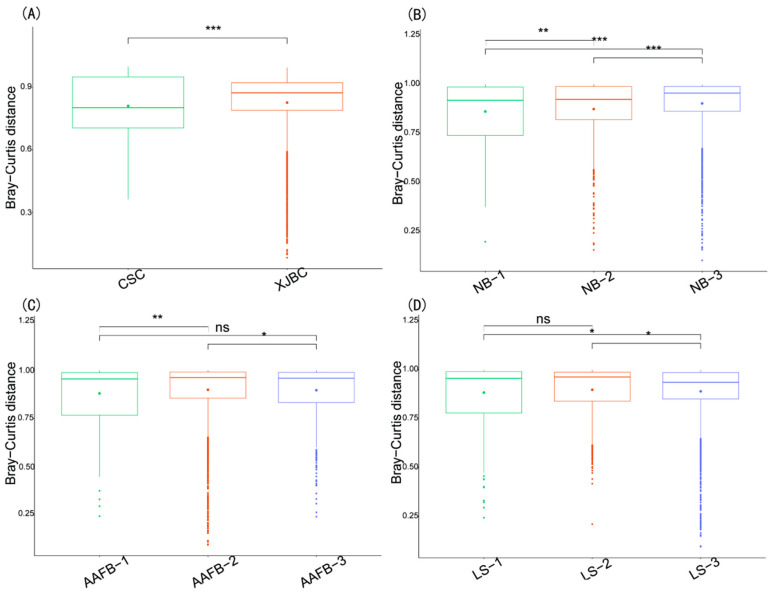
β Diversity estimation of microbial communities in the milk of dairy cows: (**A**) indicates different breeds; (**B**) indicates different parities; (**C**) indicates different first calving months of age; (**D**) indicates different stages of lactation. ns denotes not significant, “*” denotes significant, “**” denotes highly significant, and “***” denotes very significant.

In this study, we analyzed the differences in microbial community composition in milk samples from XJBC and CSC using PCoA and β diversity analysis. As shown in Figure 3 and Figure 4, the results indicated that the microbial community points in XJBC milk were more dispersed, reflecting greater microbial diversity and heterogeneity. In contrast, the microbial community points in CSC milk were more clustered, indicating lower microbial diversity. The small overlap between the microbial community points of the two breeds further emphasized the significant differences in microbial composition between XJBC and CSC milk.

The box plot results revealed highly significant microbial interactions between the two breeds. Furthermore, we categorized XJBC and CSC of different first lactation ages into three levels and conducted PCoA. The results revealed that the microbial community points for cows in the first level were more concentrated across different lactation stages. In contrast, the points for cows in the second and third levels were more dispersed, indicating substantial changes in microbial community composition. The box plot results demonstrated highly significant differences in microbial composition between the first and second levels and significant differences between the first and third levels.

We further examined the microbial community structures across different lactation stages. The findings were consistent with those for the first lactation month stages: the microbial community points were more concentrated at the first level, whereas those at the second and third levels were more dispersed. The box-and-line plot results revealed significant differences in microbial composition between the first and second levels, as well as between the first and third levels.

Finally, we categorized the cows into three levels based on different parities and performed PCoA. The results revealed that the microbial community points at the second level were more concentrated, whereas those at the first and third levels were more dispersed. These findings suggest that parity is also an important factor influencing the composition of the milk microbiome. The box plot results further revealed highly significant differences in microbial composition between the third and first levels, as well as between the third and second levels, and significant differences between the first and second levels.

The following is a refined version of the section with improved grammar, structure, and clarity.

### 3.4. Correlation Analysis of Milk and Hindgut Microorganisms in XJBC and CSC with Milk Quality and Somatic Cell Counts

**Figure 5:**
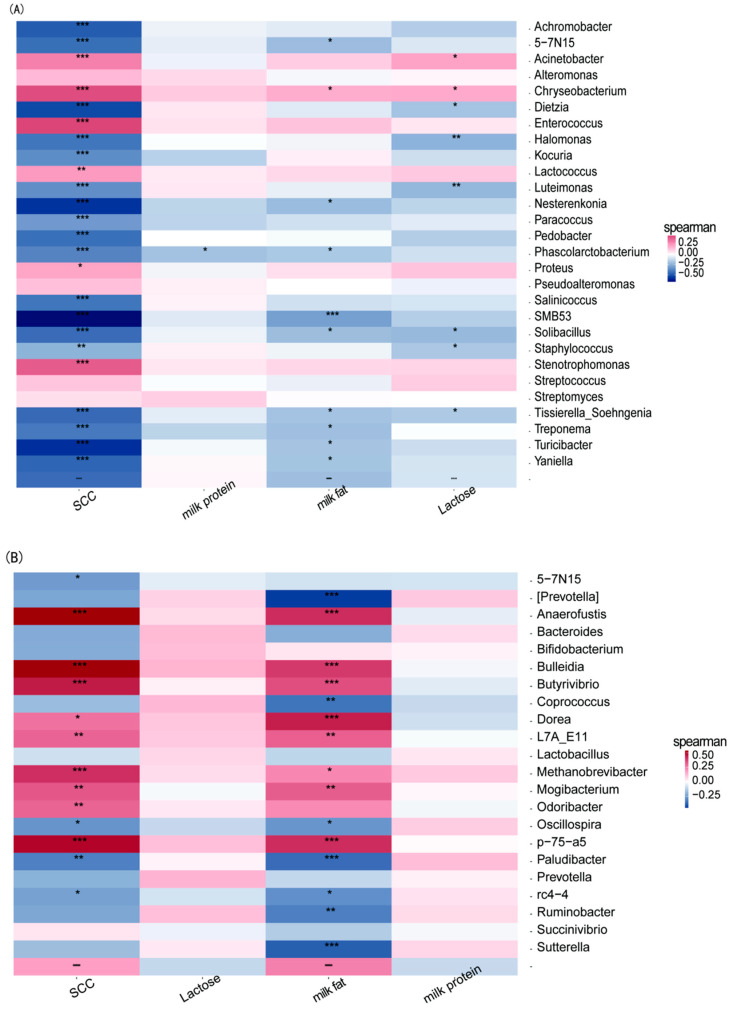
Correlation analysis of milk and hindgut microorganisms with milk fat, milk protein, lactose and somatic cell counts in dairy cows. (**A**) Milk microorganisms and (**B**) hindgut microorganisms. “*” denotes significant, ‘**’ denotes highly significant and ‘***’ denotes very significant.

As shown in Figure 5, we performed a Spearman correlation analysis of microbial genera in the milk of XJBC and CSC to explore their relationships with milk fat, protein, lactose content, and somatic cell count (SCC).

#### Milk Composition

Milk Protein: A highly significant negative correlation was observed between the abundance of the genus *Phascolarctobacterium* and milk protein content, suggesting that *Phascolarctobacterium* may inhibit milk protein production.

Milk Fat: Several genera, including *Solibacillus*, *Tissierella*, *5-7N15*, *Nesterenkonia*, *Soehngenia*, *Treponema*, *Toricibacter*, and *Yaniella*, were significantly negatively correlated with milk fat production. In addition, the genus *SMB53* also showed a significant negative correlation, indicating that these genera may collectively inhibit milk fat synthesis. Conversely, *Aspergillus chrysosporium* was highly significantly positively correlated with milk fat production, suggesting that it may promote milk fat synthesis.

Lactose: The genera *Dietzia*, *Solibacillus*, *Staphylococcus*, and *Tissierella_Soehngenia* presented significant negative correlations with lactose production. Furthermore, *Halomonas* and *Luteimonas* exhibited highly significant negative correlations, suggesting that these genera may inhibit lactose synthesis.

Somatic Cell Count (SCC): The genus *Staphylococcus* exhibited a highly significant negative correlation with SCC, suggesting its potential role in inhibiting SCC increases. A wide range of genera, including *Achromobacter*, *5-7N15*, *Dietzia*, *Halomonas*, *Kocuria*, *Luteimonas*, *Nesterenkonia*, *Paracoccus*, *Pedobacter*, *Phascolarctobacterium*, *Salinicoccus*, *SMB53*, *Solibacillus*, *Tissierella_Soehngenia*, *Treponema*, *Toricibacter*, and *Yaniella*, also showed highly significant negative correlations with SCC. In contrast, the genus *Aspergillus* was significantly positively correlated with SCC, whereas *Lactococcus* was highly significantly positively correlated with SCC. Additionally, *Fusobacterium*, *Typhimurium*, *Enterococcus*, and *Staphylococcus* exhibited highly significant positive correlations with SCC, indicating their potential contributions to increased SCC.

Hindgut Microorganisms: We also performed Spearman correlation analysis on microbial genera in the hindgut of XJBC and CSC to investigate their relationships with milk composition.

Milk Fat Production: The genera *Oscillospira* and *rc4-4* were significantly negatively correlated with milk fat production. Similarly, *Coprococcus* and *Ruminobacter* were highly significantly negatively correlated, as were *Prevotella*, *Paludibacter*, and *Sutterella*. These results suggest that these genera may inhibit milk fat synthesis. Conversely, *Methanobrevibacter* was significantly positively correlated with milk fat production. The genera *L7A_E11* and *Mogibacterium* were highly significantly positively correlated, whereas *Anaerofustis*, *Bulleidia*, *Butyrivibrio*, *Methanobrevibacter*, and *p-75-a5* were strongly positively correlated with lactolipogenesis, indicating their potential to promote milk fat production.

Somatic Cell Count (SCC): The genera *5-7N15* and *rc4-4* presented significant negative correlations with SCC, whereas *Paludibacter* was highly significantly negatively correlated with SCC, suggesting that these genera may suppress increases in SCC. Conversely, the genus *Dorea* was significantly positively correlated with the SCC. Additionally, the genera *L7A_E11*, *Mogibacterium*, and *Odoribacter* presented highly significant positive correlations, and *Anaerofustis*, *Bulleidia*, *Butyrivibrio*, *Methanobrevibacter*, and *p-75-a5* were strongly positively correlated with SCC, suggesting that their roles in contributing to SCC were increased.

### 3.5. Posterior Gut Microbes of XJBC and CSC

**Figure 6:**
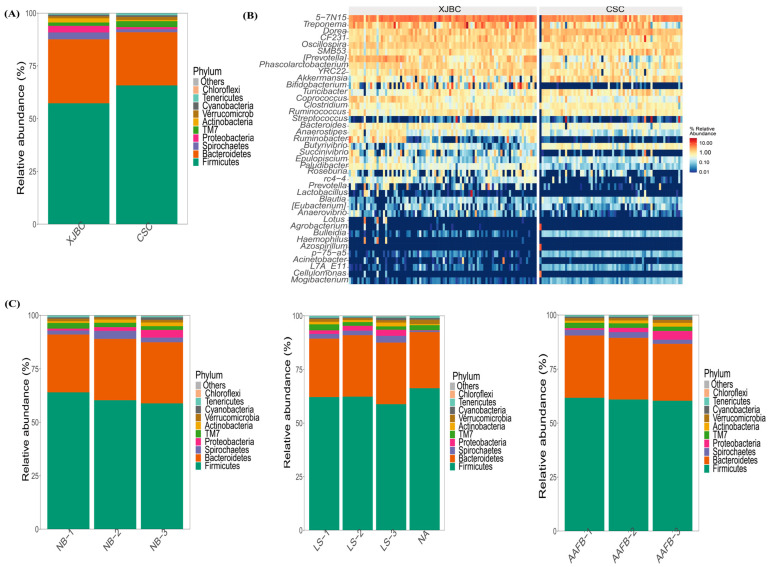
Microbiological differences in gut microbes and nongenetic factors after XJBC and CSC. (**A**) Differences in gut microbes in XJBC and CSC at the genus level and (**B**) differences in microbes at the genus level between XJBC and CSC. (**C**) Effects of nongenetic factors on gut microbes in XJBC and CSC. LS: indicates different lactation stages; AAFB: indicates different first calving months of age; NB: indicates different litter sizes.

As shown in Figure 6, the composition of the top 10 species with the highest abundance values at the phylum level in each sample was visualized using relative abundance histograms. The hindgut samples from the two breeds (XJBC and CSC) differed significantly in their bacterial community structure. Across all the samples, five phyla with relative abundance values greater than 1% were identified: *Firmicutes*, *Bacteroidetes*, *Spirochaetes*, *TM7*, and *Proteobacteria*. Together, these phyla accounted for 85% of the total abundance.

Notably, *Firmicutes* and *TM7* presented the highest abundance values in CSC, whereas *Proteobacteria* and *Spirochaetes* were most abundant in XJBC. Additionally, the cows were classified into three levels based on different first calving ages, stages of lactation, and parities, and the effects of these factors on the microbial community structure were analyzed.

First Calving Age: The results revealed that *TM7* had the highest abundance at the first level, whereas *Proteobacteria* had the highest abundance at the third level.

Lactation Stages: At different lactation stages, *TM7* presented the highest abundance at the first level, *Firmicutes* presented the highest abundance at the second level, and *Spirochaetes* and *Proteobacteria* were the most abundant at the third level.

Parity (Litter Levels): For different parities, *Firmicutes* and *TM7* presented the highest abundance at the first level, *Spirochaetes* at the second level, and *Proteobacteria* at the third level.

A heatmap of relative abundance values was also generated to illustrate the composition of the top 40 microbial species at the genus level in each sample. The heatmap provided an intuitive visualization, with a gradient of blue to red indicating an increase in the relative abundance of a microorganism. The experimental results revealed significant differences in the bacterial community structure of the hindgut samples from XJBC and CSC at the genus level. Specifically, microorganisms such as *5-7N15*, *Treponema*, *Dorea*, *CF231*, *Oscillospira*, *SMB53*, *Phascolarctobacterium*, *Prevotella*, *Akkermansia*, *YRC22*, *Turicibacter*, *Bifidobacterium*, *Coprococcus*, *Clostridium*, *Ruminococcus*, and *Bacteroides* presented relatively high relative abundance values. Among these genera, the eight hindgut microbial genera with the highest relative abundances were *Prevotella*, *Phascolarctobacterium*, *SMB53*, *Oscillospira*, *CF231*, *Dorea*, *Treponema*, and *5-7N15*.

### 3.6. α Diversity of Hindgut Microorganisms in XJBC and CSC

**Figure 7:**
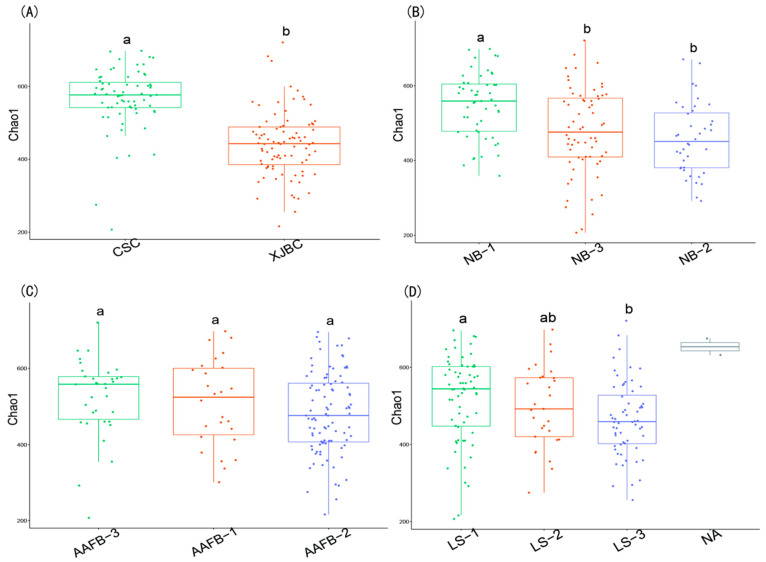
α diversity estimates of hindgut microbial communities in dairy cows. (**A**) Different breeds; (**B**) different parities; (**C**) different months of first calving age; (**D**) different lactation stages. Different lowercase letters indicate that the same lowercase letters are not significantly different.

In this study, the Chao1 index was used to measure microbial diversity and species richness in the hindgut samples of XJBC and CSC. As shown in Figure 7, the microbial diversity in the hindgut of XJBC was generally lower than that in the hindgut of CSC, with this difference being statistically significant (*p* < 0.05).

By categorizing XJBC and CSC into three levels based on their first calving age, we observed that the structure of the microbial community was relatively stable across the levels. Notably, the abundance values were significantly greater at the second level than at the third level (*p* < 0.05).

Similarly, when the cows were divided into three levels based on different lactation stages, a stable bacterial community structure was observed. The α-Chao1 index was significantly greater at the second level than at the third level (*p* < 0.05).

Additionally, when the cows were classified into three levels based on parity, the microbial community structure demonstrated similar stability. At the first level, the α-Chao1 index was significantly lower than those at the second and third levels (*p* < 0.05).

### 3.7. Results of β Diversity Analyses of Hindgut Microorganisms in XJBC and CSC

In this study, principal coordinate analysis (PCoA) was used to investigate the differences in microbial community composition in the hindgut of XJBC and CSC. This analysis aimed to explore the β diversity of hindgut microbial communities between XJBC and CSC in China.

**Figure 8:**
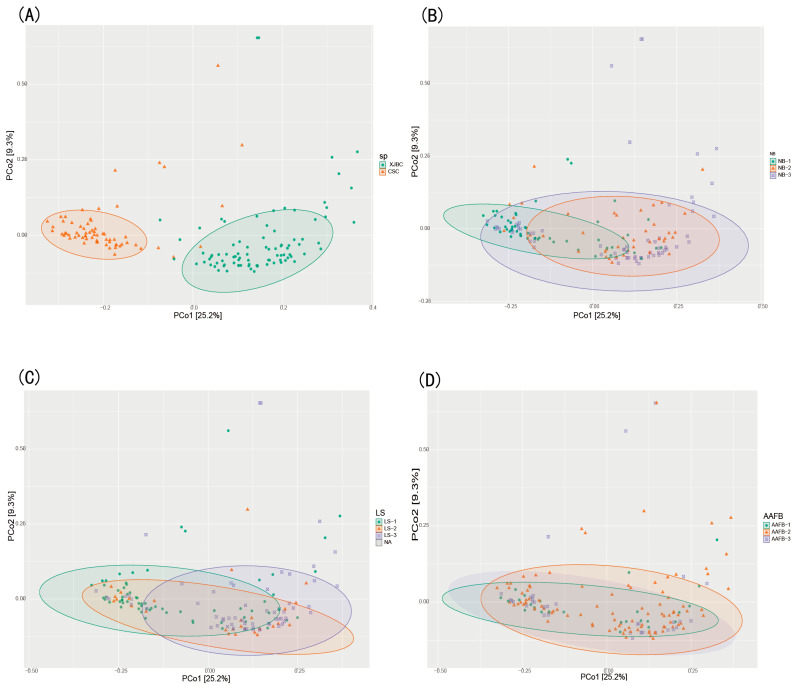
PCOA map of posterior intestinal microbes in dairy cows. (**A**) Different breeds; (**B**) different parities; (**C**) different lactation stages; (**D**) different first calving months of age.

**Figure 9:**
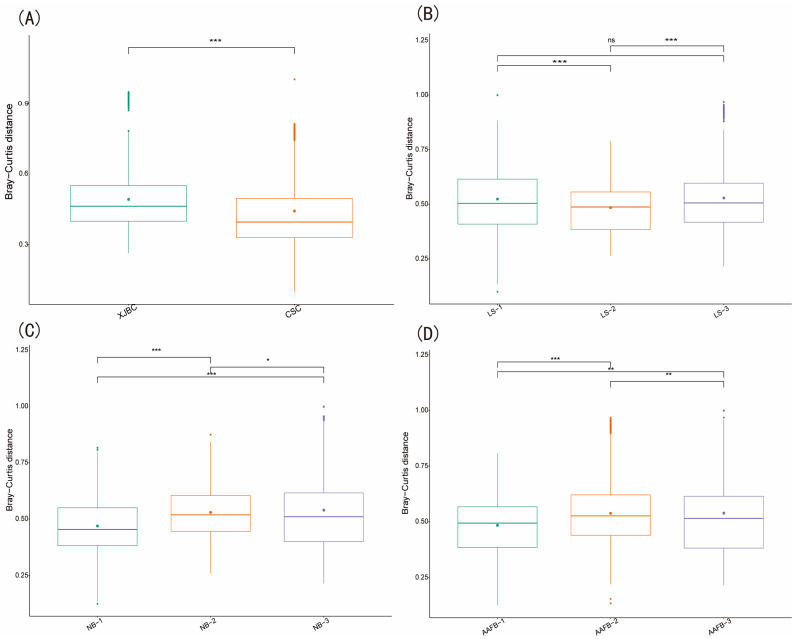
β diversity estimates of hindgut microbial communities in dairy cows. (**A**) Different breeds; (**B)** different lactation stages; (**C**) different parities; (**D**) different first calving months of age. ns denotes not significant, ‘*’ denotes significant, ‘**’ denotes highly significant, and ‘***’ denotes very significant.

As shown in Figure 8 and Figure 9, the distribution of microbial community points in the hindgut of XJBC was relatively dispersed, indicating greater variability, whereas the distribution of points in CSC was more concentrated. The box plot results confirmed significant differences in the structure of the hindgut microbial communities between the two breeds.

Effect of First Calving Age: When the cows were categorized into three levels based on their first calving age, the scatter plot revealed that the microbial community distribution at the second level was more dispersed than those at the first and third levels. The box plot results revealed significant differences among the three levels of age at first calving.

Effect of Lactation Stage: In the analysis of the lactation stage, the scatter plot revealed that microbial community points at the first level were more dispersed, whereas those at the second and third levels were more concentrated. Significant differences were observed between the first level and the second and third levels. Box plot results further revealed significant differences between the first and third levels of the lactation stage.

Effect of Parity: In the analysis of parity (number of fetuses), the scatter plot indicated that the microbial community distribution at the second level was more concentrated, whereas that at the third level was more dispersed. Compared with those at the second level, the distribution points at the third level were less clustered and showed greater variability. Box plot results highlighted significant differences in microbial communities among the three parity levels. The difference between the first and second parities was highly significant, whereas the difference between the second and third parities was significant.

### 3.8. Differential Microorganisms in the Milk of XJBC and CSC

**Figure 10:**
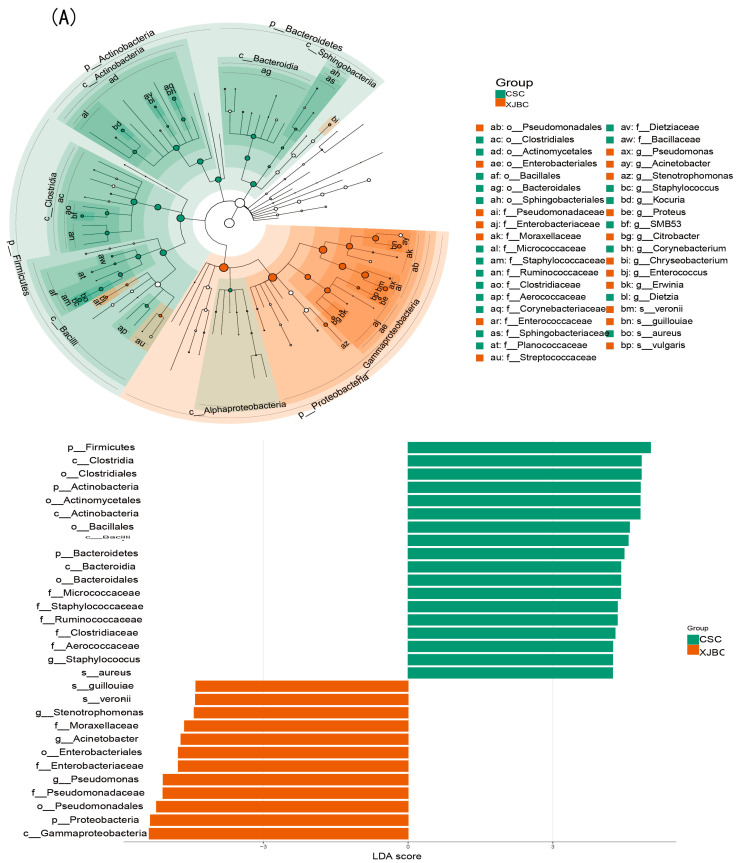

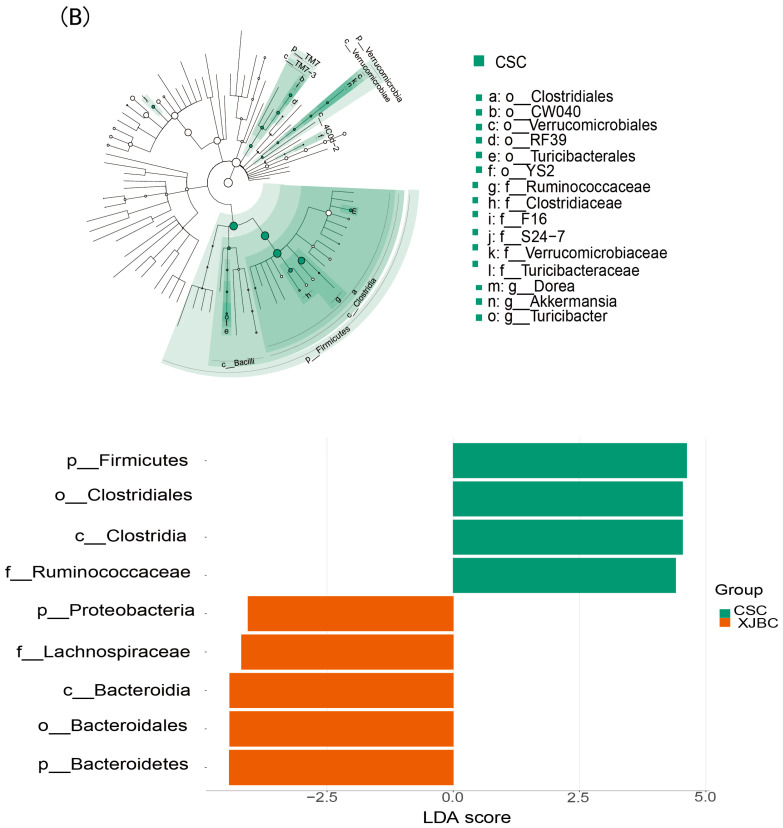
Effect size (LEfSe) analysis of linear discriminant analysis samples. Histograms of the distribution of LDA values for milk samples (LD < 4). (**A**) LEfSe branching plot of microorganisms in milk. (**B**) LEfSe branching plot of microorganisms in the hindgut.

In this experiment, as shown in Figure 10, we applied linear discriminant analysis effect size to further explore the differences in microorganisms in the milk of XJBC and CSC. At the genus level, we found that microorganisms, such as *Staphulococcus*, *Stenotrophomonas*, *Acinetobacter* and *Pseudomonas*, in the XJBC milk samples were significantly enriched. At the individual level, the microorganisms significantly enriched in the XJBC milk samples included *Pseudomonadaceae*, *Enterobacteriaceae*, *Moraxellaceae*, *Xanthomonadaceae*, *Enterococcaceae*, and *Streptococcaceae*. The microorganisms significantly enriched in the CSC milk samples included *Micrococcaceae*, *Staphylococcaceae*, *Ruminococcaceae*, *Clostridiaceae* and *Aerococcaceae*. Microorganisms with relatively high abundances in the hindgut of CSC included *p_Firmicutes*, *o_Clostridiales*, *c_Clostridia f_Ruminococcaceae*, *p_Proteobacteria*, *f_Lachnospiraceae*, *C_Bacteroidia*, *o_Bacteroidales*, and *p_Bacteroidetes*. In LEfSe multilevel species-level analysis, the intestinal samples of Simmental cattle in China were significantly enriched with microorganisms such as *Ruminococcaceae*, *Clostridiaceae*, *F16*, *Verrucomicrobiaceae*, *Akkermansia*, *Dorea* and *SMB53*.

### 3.9. Milk Microorganisms and Hindgut Microorganisms in XJBC and CSC

**Figure 11:**
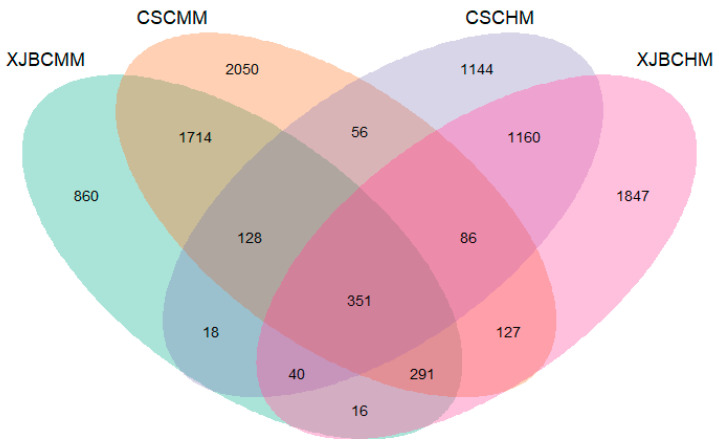
Venn diagrams of the OTUs of different groups of XJBC milk microorganisms (XJBCMMs), XJBC hindgut microorganisms (XJBCHMs), CSC milk microorganisms (CSCMMs) and CSC hindgut microorganisms (CSCHMs).

In this study, we compared the microbial composition in the milk of XJBC and CSC and their respective hindgut microbial communities by means of Venn diagram analysis, as shown in Figure 11. The visualization of the Venn diagram reveals the overlap of operational classification units (OTUs) between different samples. These data revealed that despite the presence of some shared microbiota, there were significant differences in microbial diversity between samples, with 860 OTUs specific to XJBC milk and 2050 to CSC milk and 1144 in the hindgut of XJBC compared with 1847 in the hindgut of CSC.

### 3.10. Characterization of Microflora in the Hindgut and Milk of XJBC and CSC

In this study, a total of 342 microorganisms were selected for functional labeling through repeated microbial screening of the common flora in the milk and hindgut of XJBC and China Simmental cattle. Specifically, 62 kinds of microorganisms related to digestion and metabolism and milk composition were selected from these microorganisms for in-depth analysis. The functional annotation of these microbes is critical for understanding their potential roles in milk and the hindgut. For example, some specific microorganisms may be involved in the synthesis of milk proteins, milk lipids and lactose, which are key nutritional indicators for assessing the nutritional value of milk. Gastroenter–liver–mammary gland synergism plays an important role in milk component synthesis, which is regulated by various signaling pathways. Gaining insight into the functions of these microorganisms could inform nutrient regulation strategies to improve milk composition. In addition, by analyzing the abundance values of these microorganisms, it is possible to reveal their roles in different breeds and different physiological stages of cattle. For example, certain microorganisms may play more important roles in specific stages of milk production or parity in cows, which may be related to digestive health, nutrient absorption and disease resistance.

### 3.11. Association Analysis Between Milk and Hindgut Microorganisms in XJBC and CSC

As shown in Schedules S1 and S2, this experiment explored the associations between milk and hindgut microorganisms in parturient cows using three statistical methods: the chi-square test, likelihood ratio, and Fisher’s exact test.

Significant Correlations in CSC: Highly significant correlations (*p* < 0.001) were detected for the following microorganisms in the milk and hindgut of CSC: *Prevotella*, *Akkermansia*, *Blautia*, *Coprococcus*, *Faecalibacterium*, *Ruminococcus*, *Nitrospira*, *Klebsiella*, *Oscillospira*, *Dorea*, *Dialister*, *Cetobacterium*, *Lactobacillus*, *Anaerovibrio*, *Syntrophomonas*, *Butyricimonas*, *Odoribacter*, *Veillonella*, *Butyrivibrio*, and *Bifidobacterium*, among others.

### 3.12. Association Analysis Between Mid-Milk and Postintestinal Microorganisms in XJBC and CSC

As shown in Schedules S1 and S2, this study explored the associations between microorganisms and hindgut microorganisms in the milk of parturient cows using three different statistical methods: the chi-square test, likelihood ratio, and Fisher’s exact test. The following correlations between microorganisms and hindgut microorganisms in the milk of CSC were highly significant (*p* < 0.001): *Prevotella*, *Akkermansia*, *Blautia*, *Coprococcus*, *Faecalibacterium*, *GOUTA19*, *Eubacterium*, *Ruminococcus*, *Nitrospira*, *Klebsiella*, *Oscillospira*, *Dorea*, *Dialister*, *Cetobacterium*, *Enterococcus*, *Lactobacillus*, *Anaerovibrio*, *Syntrophomonas*, *Butyricimonas*, *Odoribacter*, *Citrobacter*, *Veillonella*, *Parabacteroides*, *Butyrivibrio*, *Proteiniclasticum*, *Megamonas*, *Collinsella*, *Bulleidia*, *Catenibacterium Phascolarctobacterium*, *Epulopiscium*, *Lactococcus*, *Paludibacter*, *Lachnospira*, *Turicibacter*, *Steroidobacter*, *Succinivibrio*, *Megasphaera*, *Acetobacter*, *Hylemonella*, *Ruminobacter*, *Pediococcus*, *Brachybacterium*, *Bifidobacterium*, *Bilophila*, *Pseudaminobacter*, *Brevundimonas* and *Mucispirillum*. Moreover, the following highly significant *(p* < 0.001) correlations between microorganisms and hindgut microorganisms in the milk of XJBC were detected*: Prevotella*, *Blautia*, *Bacteroides*, *Coprococcus*, *Ruminococcus*, *Eubacterium*, *Ruminococcus*, *Nitrospira*, *Klebsiella*, *Dorea*, *Cetobacterium*, *Sutterella*, *Lactobacillus*, *Syntrophomonas*, *Butyricimonas*, *Odoribacter*, *Proteiniclasticum*, *Allobaculum*, *Megamonas*, *Phascolarctobacterium*, *Epulopiscium*, *Paludibacter*, *Turicibacter*, *Hyle Salmonella*, *Ruminobacter*, *Brachybacterium*, *Bifidobacterium*, *Gluconacetobacter*, and *Pseudaminobacter*. There were highly significant associations between milk microorganisms and hindgut microorganisms shared by XJBC and CSC for *Prevotella*, *Coprococcus*, *Ruminococcus*, *Eubacterium*, *Nitrospira*, *Klebsiella*, *Dorea*, *Cetobacterium*, *Lactobacillus*, *Syntrophomonas*, *Butyricimonas*, *Odoribacter*, *Proteiniclasticum*, *Megamonas*, *Phascolarctobacterium*, *Epulopiscium*, *Paludibacter*, *Turicibacter*, *Hylemonella*, *Ruminobacter*, *Brachybacterium*, *Pseudaminobacter* and *Bifidobacterium*.

## 4. Discussion

### 4.1. Characterization of Microorganisms in XJBC and CSC Milk

To explore the diversity of microorganisms in XJBC and CSC milk, milk samples were analyzed in depth using 16S rRNA sequencing in this study. The results revealed significant differences in the microbial community composition between the milk samples from the two cow breeds. At the phylum level, there were five microbial phyla with relative abundances exceeding 1%, namely, *Proteobacteria*, *Firmicutes*, *Bacteroidetes*, *Actinobacteria* and *Tenericutes*. These findings are similar to those of previous studies conducted on milk samples from Holstein cows [26].

These findings suggest that milk microbial communities may be common between different regions and breeds of cattle. The genus *Proteobacteria* includes many pathogenic microorganisms that increase the risk of the mammary gland suffering from mastitis [27], whereas the genus *Firmicutes* produces lactic acid, which maintains an acidic environment and thus acts as an inhibitor of harmful flora [28]. Significant differences (*p* < 0.05) were observed in the microorganisms between the species. At the genus level, these three genera, namely, *Pseudomonas*, Lactobacillus, and *Corynebacterium*, are important in the microbial community in both types of cow’s milk. *Pseudomonas*, bacteria of this genus, are known for their wide range of environmental adaptation and metabolic diversity, and their metabolism, and thus the production of toxins, causes inflammation in the mammary gland [29]. Lactobacillus plays a very important role in food fermentation and produces lactic acid and other metabolites to inhibit the growth of pathogenic microorganisms and maintain the intestinal flora balance [12]. The genus *Corynebacterium* has a wide range of environments in which it plays a beneficial role in the production and processing of dairy products and may compete with pathogenic bacteria, thereby reducing the incidence of mastitis [30]. The species level of *Corynebacterium bovis* has been associated with subclinical mastitis in cow’s milk [31]. At the species level, between XJBC and CSC, the highest relative abundance of *Proteobacteria* was found in the milk of XJBC, whereas the highest relative abundance of *Firmicutes* was found in CSC, and the mammary gland health status of XJBC was lower than that of CSC, which should be strengthened by the feeding management of XJBC on this farm.

### 4.2. Influence of Microorganisms in Milk on the Lactation Performance of XJBC and CSC

In this study, we performed Spearman’s correlation analysis of microbial genera in the hindgut and milk of XJBC and CSC to investigate the relationships between these microorganisms and milk lipids, milk proteins, and lactose. The analysis of the results revealed that the microbial genera in milk were significantly correlated with the production of milk components. In this study, we found that *Phascolarctobacterium*, *Solibacillus*, *Tissierella*, *5-7N15*, *Nesterenkonia*, *Phascolarctobacterium*, *Soehngenia*, *Treponema*, *Turicibacter*, *Yaniella*, and *SMB53* had inhibitory effects on lactolipid production. *Lactose inhibition by Dietzia*, *Solibacillus*, *Staphylococcus*, *Tissierella_Soehngenia*, *Halomonas*, and *Luteimonas*. *Acinetobacter* and *Chryseobacterium* had highly significant positive correlations with milk fat production. Previous studies revealed that the relative abundance values of some of these flora changed during the development of mastitis in dairy cows [32], and these flora may induce mastitis in dairy cows through estrogen-, lipid metabolism-, immune regulation- and inflammation-related responses, thus reducing milk quality [33]. Previous studies have shown that the *SMB53* genus of microorganisms is ubiquitous in cow’s milk, and its function and significance in the mammary gland need to be further investigated [34]. In this study, we found that this genus of bacteria positively regulates milk fat production in cows, and the specific mechanisms should be further explored. Previous studies have shown that *Acinetobacter* species are involved in digestive metabolism in the gastrointestinal tract [35], and in the present study, *Acinetobacter* positively regulated lactose production, which may be related to the gut–mammary pathway, possibly because the relatively high relative abundance values of the genus in the gastrointestinal tract increase feed conversion and thus lactose, and the genus may invade the mammary tissue exogenously or endogenously. *Staphylococcus*, *Achromobacter*, *5-7N15*, *Dietzia*, *Halomonas*, *Kocuria*, *Luteimonas*, *Nesterenkonia*, *Paracoccus*, *Pedobacter*, *Phascolarctobacterium*, *Salinicoccus*, *SMB53*, *Solibacillus*, *Tissierella_Soehngenia*, *Treponema*, *Turicibacter*, and *Yaniella* were highly significantly negatively correlated with somatic cell counts and decreased somatic cell counts in the milk of lactating cows. The levels of the microbial genera Proteus, *Lactococcus*, *Acinetobacter*, *Chryseobacterium*, and *Enterococcus* were highly significantly positively correlated with the number of somatic cells, increasing the number of somatic cells in the milk of lactating cows. High somatic cell counts in dairy cows may affect the state of mammary gland health and influence milk production and milk composition [36]. A previous study revealed that the predominant groups associated with subclinical mastitis were *Proteobacteria*, *Bacteroidetes*, *Firmicutes*, and *Actinobacteria*, and the predominant genera associated with *Firmicutes* were *Streptococcus*, *Enterococcus*, *Staphylococcus*, and *Bacillus* [37]. The results of this study need to be further investigated. Spearman’s correlation analysis of the effects of microorganisms on milk quality and the somatic cell count in the hind milk of lactating cows revealed significant negative correlations between the flora *Turicibacter* and *Phascolarctobacterium* and milk fat and very significant negative correlations with the somatic cell count. Previous studies have shown that the flora *Turicibacter* and *Phascolarctobacterium* have high abundance values in milk, blood and the intestine [38]. The effects of microorganisms in the mammary glands of cows on milk quality need to be further verified.

### 4.3. Characteristics of Microorganisms in the Hindgut Contents of XJBC and CSC and Their Effects on Lactation Performance

Among all the samples, five phyla had relative abundance values greater than 1%, including *Firmicutes*, *Bacteroidetes*, *Spirochaetes*, *TM7* and *Proteobacteria*, which accounted for 85% of the total abundance values. Firmicutes and TM7 had the highest abundance values in CSC, whereas *Proteobacteria*, *Spirochaetes* and *Proteobacteria* had the highest abundance values in XJBC. The results of this study are similar to those of previous studies; the main flora were Firmicutes, *Bacteroidetes* and *Proteobacteria* [39]. Previous studies have shown that the main functions of the genus Firmicutes are related to carbohydrate metabolism, amino acid metabolism and transcriptional repair functions [40]. A previous study revealed that the abundances of *Bacteroidetes*, *Spirochaetes* and *Proteobacteria* were greater in the concentrate feed 4 L milk yield group than in the 2 L milk yield group under high-concentrate feed conditions, and the results indicated that these flora may contribute to milk production in dairy cows. TM7 abundance values were greater in the medium-concentrate feed group than in the high-concentrate feed group [41]. The gastrointestinal tract can be stressed by a variety of stimuli, thus leading to changes in the microflora in the gut, such as lack of feed [42], changes in dietary habits [43], and the heat stress response [44].

The gut microbiota plays many important roles in the digestive process. Milk yield and composition are influenced by genetic, nutritional, physiological and environmental factors. In particular, nutrition has a very strong influence on the composition of milk fat [45], whereas the composition of the protein fraction is usually only slightly influenced by this factor; in fact, the fatty acids in milk are almost equally derived from nutrition (long fatty acids) and synthesized from scratch (short fatty acids) [46]. The results of the analysis revealed significant correlations between specific microbial genera and milk composition at the genus level.

Specifically, Oscillospira, rc4-4, Coprococcus, Ruminobacter, Prevotella, Paludibacter, and Sutterella were negatively correlated with milk fat production. Methanobrevibacter, L7A_E11, Mogibacterium, Anaerofustis, Bulleidia, Butyrivibrio, Methanobrevibacter, and p-75-a5 were positively associated with milk fat production. Previous studies and the results of the present study were similarly negatively correlated with milk fat production, and the abundance of Prevotella decreased during the breast milk phase in female animals but became the main dominant flora during the weaning period [47]. A previous study revealed negative correlations between two microorganisms, Crococcus and Ruminobacter, and milk yield and milk quality, which is in agreement with the findings of the present study [48]. For Butyrivibrio, Dorea, Anaeroplasma, Sutterella and Paludibacter, the results of previous studies revealed significant enrichment of these microorganisms in the milk of high-yield cows, and in the present study, these microorganisms were negatively correlated with the production of milk fat, which may be the case when milk production increases, leading to a decrease in the milk fat percentage [49]. 5-7N15, rc4-4, and Paludibacter were significantly negatively correlated with the somatic cell count, whereas the levels of Dorea, L7A_E11, Mogibacterium, Odoribacter, Anaerofustis, Bulleidia, Butyrivibrio, Methanobrevibacter and p-75-a5 were highly significantly and positively correlated with somatic cell counts. Spearman’s correlation analysis of the effects of hindgut microorganisms on milk quality and somatic cell counts in lactating cows revealed negative correlations between the abundances of the organisms Prevotella, Coprococcus, Paludibacter and Ruminobacter and milk fat in lactating cows.

There was an extremely significant negative correlation with the somatic cell count of Paludibacter and significant positive correlations with the somatic cell count in *Dorea* and *Odoribacter*. *Prevotella*, *Coprococcus*, *Dorea*, *Odoribacter*, *Paludibacter*, *Turicibacter* and *Ruminobacter* were present in both the milk and the hindgut and were correlated (*p* < 0.001). Previous studies have shown that *Prevotella*, *Coprococcus*, *Turicibacter*, and *Ruminobacter* are mainly responsible for digestive metabolism in the ruminant gut [50]. Exogenous microbial invasion disrupts the balance of the mammary flora through the entry of microorganisms from the environment into the mammary gland through damaged mammary tissue and the unsealed teats of cows [13]. The results of the present study revealed very significant correlations between microorganisms in cow’s milk and microorganisms in the hindgut. Reasonable feed ratios can reduce the incidence of mastitis in parturient cows, and from the point of view of microbial invasion of the mammary gland, maintaining a good feeding environment, treating feces regularly, and paying attention to the milking method are recommended to reduce damage to the teats of parturient cows and to reduce the incursion of exogenous invasions into the mammary gland.

### 4.4. Microbial Associations in the Hindgut and Milk of XJBC and CSC

The possible sources of microorganisms in the milk of cows are categorized as follows: genetically determined [51], invasion of the mammary gland by microorganisms in the environment through damaged teats [52], increased diversity of microorganisms in the milk of cows delivered vaginally [53], and the endogenous pathway, which refers to the migration of gut microorganisms of the intestinal tract of the gut–mammary pathway toward the mammary gland through a specific mechanism [54]. In this study, the microbiota composition of the samples was analyzed using 16S rRNA gene sequencing to reveal the associations between microorganisms in milk and hindgut organisms of parturient cows. First, 351 microorganisms common to the milk and hindgut of XJBC and CSC were functionally annotated, and 61 flora related to digestive metabolism and milk composition were selected as the microorganisms in the milk and the microorganisms in the hindgut of XJBC, respectively. Correlation analysis was performed to select 23 highly significant (*p* < 0.001) microbial groups in the milk and the hindgut between the two breeds: *Prevotella*, *Coprococcus*, *Ruminococcus*, *Eubacterium*, *Nitrospira*, *Klebsiella*, *Dorea*, *Cetobacterium*, *Lactobacillus*, *Syntrophomonas*, *Butyricimonas*, *Odoribacter*, *Proteiniclasticum*, *Megamonas*, *Phascolarctobacterium*, *Epulopiscium*, *Paludibacter*, *Turicibacter*, *Hylemonella*, *Ruminobacter*, *Brachybacterium*, *Pseudaminobacter* and *Bifidobacterium*. Previous studies have shown that natural birth leads to an increase in the diversity of microorganisms in the mammary gland. Rational means of delivery can reduce the probability of mastitis. The results of previous studies have shown that the main phylum-level bacteria that enter the mammary gland through the intestinal and mammary pathways are *Proteobacteria*, *Bacteroidetes*, *Firmicutes*, *Actinobacteria*, *Fusobacteria*, and *Tenericutes*, as well as *Acinetobacter*, *Campylobacter*, *Bacillus*, *Enterobacter*, *Staphylococcus*, *Streptococcus* and *Kocuria* [55,56]. In addition, our results echo those of previous studies by confirming that intestinal microorganisms are able to migrate to the mammary gland through specific mechanisms. This study provides not only a new perspective for understanding the origin and mechanism of action of microorganisms in the milk of dairy cows but also a scientific basis for improving the health of dairy cows and enhancing milk quality. Future studies can further explore the functional roles of these specific flora and how they affect cow health and milk quality through the gut–mammary pathway.

### 4.5. Influence of Nongenetic Factors on Intestinal Microbial Changes During and After Lactation in XJBC and CSC

In this study, the changes in the *microflora* in the milk of XJBC and CSC at different first calving months of age, lactation and parity were studied, and the physiological changes in the cows themselves also led to changes in the *microflora* in milk [57,58]. Regarding the age of first calving, the microbial diversity of the third stage was significantly greater than that of the first and second stages. *Firmicutes* and *Actinomycetes* were the most abundant bacteria in the first stage, and *Proteobacteria* were the most abundant bacteria in the second stage (*p* < 0.05). The microbial diversity in milk increased with increasing lactation age. The diversity in the third level of lactation was significantly greater than that in the first level (*p* < 0.05). *Firmicutes*, *Actinomycetes* and *Micrococcaceae* had the highest abundances at the first level. At the third level, the abundance of *Proteobacteria* was the highest. The microbial diversity in dairy milk increased with increasing lactation time. Previous research results were consistent with these results, indicating that the mammary gland microbial diversity of dairy cows increases with increasing lactation time, which may be related to the feeding conditions of dairy cows [59]. Previous studies have shown that with increasing milking time and worker frequency, milk nipples are damaged, leading to the invasion of external microorganisms into the mammary gland [60]. The microbial diversity in the third level of milk production was significantly greater than that in the first level (*p* < 0.05). At the first level, *Firmicutes*, *Actinobacteria* and *Bacteroidetes* had the highest abundance values, whereas at the second level, *Proteobacteria* had the highest abundance values. The diversity of microorganisms in milk increases with the number of milking times. In both normal and mastitis-affected milk samples, *Actinomycetes* was the most dominant phylum. Over time, increased milk production in cows can damage lactating tissue, causing microbiota to enter and leading to mastitis infections. To maintain the health and well-being of these animals, the use of proper milking techniques and the provision of a good feeding environment are essential to minimize the incidence of mastitis and other diseases.

The effects of different months of first lactation, lactation periods, litter sizes and diets on microbial changes in XJBC and CSC were also studied, as were the effects of lactation, parity and other physiological states on changes in the intestinal flora. In this study, 16S rRNA gene sequencing was used to analyze the relationships between different first calving months of age, lactation periods and parity of cows, and significant differences were found in the composition of the microbiota in the samples. At the phylum level, five phyla presented relative abundance values greater than 1%. The relative abundance values of microorganisms at different first calving months of age were similar, and the microbial diversity at the secondary level was significantly greater than that at the tertiary level (*p* < 0.05). This may be related to differences in the feeding environment, lactation period and parity of the cows. The highest abundance value of *TM7* was detected at the first level, the highest abundance value of *Firmicutes* was detected at the second level, the highest abundance values of *Spirochaetes* and *Proteobacteria* were detected at the third level, and the microbial diversity was significantly greater at the second level than at the third level (*p* < 0.05). However, similar to the results of this study, previous studies have shown that microbes in the late stage of lactation decreased significantly with increasing lactation time compared with pre-lactation and mid-lactation [61]. The highest abundances of *Firmicutes* and TM7 in the different lactation stages were at the first level, the highest abundance of Spirochetes was at the second level, and the highest abundance of *Proteobacteria* was at the third level. The microbial diversity at the first level was significantly lower than those at the second and third levels (*p* < 0.05). Previous studies have shown that fetal delivery has a significant effect on the type and abundance of microorganisms in the hindgut [62].

In summary, the age of the first calving months, lactation stage and parity are important factors affecting the changes in the intestinal microflora of dairy cows after milk neutralization. The findings of this study highlight the importance of proper milking techniques and the provision of a good feeding environment to maintain cow health and reduce the incidence of mastitis and other diseases. Future studies should further explore the functional roles of these specific flora and how they affect the health and milk quality of dairy cows.

## 5. Conclusions

In this study, we utilized high-throughput sequencing technology to analyze the 16S rRNA genes in the V3 and V4 hypervariable regions of milk microbial communities from XJBC and CSC cattle. Through comprehensive analysis, we characterized the microbial communities in both milk and the hindgut across these two breeds. The study initially compared microbial communities at various levels, including breed, age at first calving, lactation stage, and cow parity, revealing significant differences among them. Spearman’s correlation analysis was then employed to identify key microbial species in the mammary gland and intestinal tract that significantly impact milk fat, protein, and lactose content in dairy cows.

Finally, using chi-square tests, maximum likelihood estimation, and Fisher’s exact test, we identified 23 microorganisms that were significantly correlated between milk and the gut in both breeds, with *p*-values less than 0.001. These findings provide a theoretical foundation for optimizing production practices in periparturient cows, potentially reducing the risk of mammary gland infections caused by exogenous microorganisms and ultimately lowering the incidence of mastitis in heifers.

## Figures and Tables

**Table 1:** Horizontal division descriptive statistics.

Sample Sources	Heritable Condition (i.e., Not Genetic)	Horizontal Division Range	Sample Size (Statistics)
Microorganisms in milk samples	First calving months age	20–27 months	110
		28–30 months	53
		>30 months	33
	parity	First pregnancy	47
		Second pregnancy	87
		Third pregnancy and above	62
	Stage of lactation	Calving to the end of the tenth week	71
		Eleventh week to the end of the twentieth week	45
		Twenty-first week after	78
Microorganisms in hindgut samples	First calving months age	20–27 months	33
		28–30 months	51
		>30 months	103
	parity	First pregnancy	60
		Second pregnancy	44
		Third pregnancy and above	83
	Stage of lactation	Calving to the end of the tenth week	68
		Eleventh week to the end of the twentieth week	42
		Twenty-first week after	75

Note: Sample sizes in different levels of nongenetic factor division between XJBC and CSC.

## Data Availability

The raw data including metabolome raw data, analytical codes and other collected data that support the findings of this study are available from the corresponding author upon request.

## Supplementary Materials

The following supporting information can be downloaded at: https://www.mdpi.com/article/10.3390/microorganisms13020448/s1, Schedule S1: Microbial Functional Notes; Schedule S2: Microbial chi-square test in hindgut and milk of Chinese Simmental cattle; Schedule S3: Microbial chi-square test in hindgut and milk of Xinjiang Province cattle.
