# Supplementary material for: The Effects of Milk and Posterior Intestinal Microorganisms on the Lactation Performance of Dual-Purpose Cattle (Bos taurus) Revealed by 16S rRNA Sequencing
Source: Microorganisms. 2025 Feb 18;13(2):448. doi: 10.3390/microorganisms13020448 (PMC11857882; doi:10.3390/microorganisms13020448)
Supplement: Supplementary file 1 [file microorganisms-13-00448-s001.zip › microorganisms-3448160-supplementary.pdf]

Schedule S1. Microbial Functional Notes.

Name of Microorganism	Functional Notes
Prevotella	A bacterium commonly found in the oral cavity and intestinal tract, especially in periodontitis and in the intestinal microbiota. They are involved in the breakdown of polysaccharides to produce short-chain fatty acids.
Akkermansia	Considered a beneficial bacterium associated with intestinal health and immune regulation. Its ability to break down mucus has a positive effect on maintaining intestinal barrier function.
Blautia	are bacteria that produce short-chain fatty acids (e.g., butyric acid) in the intestines, which are beneficial to intestinal health. They are also involved in anti-inflammatory processes and immunomodulation.
Bacteroides、Parabacteroides	One of the major members of the gut microbial community, involved in the breakdown of polysaccharides and the production of short-chain fatty acids. They play an important role in gut health and disease.
Coprococcus	An important genus of bacteria in the intestinal tract that ferments carbohydrates and produces butyric acid, which is beneficial to intestinal health. May help suppress the immune response and reduce the severity of allergic responses.
Faecalibacterium	is a bacterium that produces large amounts of butyric acid, which is important for gut health.Butyric acid is an important source of energy for intestinal epithelial cells and has anti-inflammatory properties.
Morganella	Usually found in the intestinal tract, but may cause infections in some cases.They are adaptable and survivable in their environment.
GOUTA19、Proteiniclasticum	This name is not common in microbial classification and may be the name used in a particular study or a mistake.
Roseburia	It is a type of bacteria that produces butyric acid in the intestines, which is beneficial for intestinal health.They are involved in anti-inflammatory processes and immune regulation.
Ruminococcus	In the intestinal microbial community, especially bacteria associated with fiber fermentation.They are involved in the breakdown of polysaccharides to produce short-chain fatty acids.
Campylobacter	Usually associated with food poisoning, they are pathogenic bacteria.Their abundance in the intestinal tract is usually low, but in some cases they may cause disease.

Klebsiella、Citrobacter	are a type of bacteria that are common in the gut but may cause infections under certain circumstances.They have a wide range of environmental adaptations and can cause disease outside the gut.
Oscillospira	are a type of bacteria commonly found in the intestinal tract and are involved in the breakdown of polysaccharides and the production of short-chain fatty acids.They play an important role in intestinal health and disease.
Dialister	in the gut microbial community are associated with oral health and intestinal inflammatory diseases. They may be involved in metabolic processes in the oral cavity and gut.
Cetobacterium	are a type of bacteria found in the intestinal tract that have been linked to intestinal health and disease. They may be involved in metabolic processes in the gut.
Sutterella	in the intestinal microbial community associated with inflammatory bowel disease. They may play a role in the development of intestinal diseases.
Enterococcus、Providencia	are a type of bacteria that are commonly found in the intestinal tract, but may cause infections in some cases. They are resistant to a wide range of antibiotics and can cause infections in hospital settings.
Leuconostoc	are a type of bacteria commonly found in fermented foods, such as kimchi and wine. They produce lactic acid, which is important for the flavor and preservation of foods.
Anaerococcus	are anaerobic bacteria usually found in the gut and on the skin. Their role in the intestinal microbial community is unknown, but they may be involved in metabolic processes.
Lactobacillus、Bifidobacterium	A probiotic commonly found in the intestines and vagina for health benefits. They produce lactic acid, which helps inhibit the growth of pathogenic bacteria and are involved in immune regulation.
Anaerovibrio	are anaerobic bacteria that are usually found in the intestinal tract. They may be involved in metabolic processes in the gut, but their exact function is unknown.
Butyricimonas、Butyrivibrio	It is a bacterium that produces butyric acid in the intestines, which is beneficial for intestinal health. Butyric acid is an important source of energy and has a protective effect on intestinal epithelial cells.
Fibrobacter	A bacterium that breaks down fiber in the intestines, producing short-chain fatty acids. They are particularly important in the digestion of ruminants.

Odoribacter、Megamonas、Collinsella、Anaerotruncus、Lachnospira	are a type of bacteria commonly found in the gut and are associated with intestinal health. They may be involved in metabolic processes in the gut.
Veillonella、Paraprevotella	A bacterium commonly found in the oral cavity and intestinal tract and associated with acid production. Their role in the intestinal microbial community is unknown, but they may be involved in metabolic processes.
Methanobrevibacter	is an archaeon that produces methane in the gut. Their role in the gut microbial community is unknown, but they may be involved in methane production and energy balance.
Actinomyces	are a type of bacteria commonly found in the oral cavity and intestinal tract and are associated with oral health. They may be involved in metabolic processes in the mouth and gut.
Propionicimonas	It is a bacterium that produces propionic acid in the intestines, which is beneficial for intestinal health. Propionic acid is an important source of energy and has a protective effect on intestinal epithelial cells.
Lactococcus	are a type of bacteria commonly found in fermented foods, such as cheese and yogurt. They produce lactic acid, which is important for the flavor and preservation of foods.
Megasphaera	are a type of bacteria commonly found in the oral cavity and intestinal tract and are associated with acid production. They are found in the gut microbiota
Peptococcus	are a type of bacteria commonly found in the oral cavity and intestinal tract and are associated with acid production. They are found in the gut microbiota
Anaerostipes	are strictly anaerobic bacteria that are usually found in the intestinal tract. They may be involved in metabolic processes in the gut
Ruminobacter	A bacterium found in the rumen of ruminants that is involved in the breakdown of fiber and helps ruminants digest plant material.
Pediococcus	are a type of bacteria commonly found in fermented foods such as sausages and sauerkraut. They produce lactic acid, which is important for the flavor and preservation of foods.
Mucispirillum	are a type of spiral-shaped bacteria found in the intestinal tract and are associated with intestinal health. They may

	be involved in metabolic processes in the gut and have been linked to the development of certain diseases.
Eggerthella	It is a bacterium that is commonly found in the gut, especially in the elderly. They are involved in the breakdown of polysaccharides and the production of short-chain fatty acids.
[Eubacterium]	They belong to the phylum Thick-walled Bacteria and are involved in the metabolism of carbohydrates and amino acids, which are beneficial for gut health. Their core functions in the gut microbiota include energy metabolism and amino acid metabolism .
[Ruminococcus]	It is one of the major members of the intestinal microbiota and is involved in the breakdown of polysaccharides and the production of short-chain fatty acids, which play an important role in maintaining intestinal health and regulating host metabolism .
[Prevotella]	In the human gut, Prevotella spp. are associated with non-Western dietary and nutritional patterns rich in carbohydrates, resistant starch and fiber.They specialize in short-chain fatty acid propionic acid from arabinoxylan and oligofructose, which are beneficial for gut health .
Allobaculum、Adlercreutzia、Bulleidia、Catenibacterium、Phascolarctobacterium、Epulopiscium、Slackia、Turicibacter、Dysgonomonas、Bilophila、Pseudoramibacter	are a type of bacteria found in the intestinal tract and are associated with intestinal health.They may be involved in metabolic processes in the gut.

Note: Microorganisms common to cow's milk and the hindgut were annotated to select microorganisms affecting milk quality and mastitis for the chi-square test.

Schedule S2. Microbial chi-square test in hindgut and milk of Chinese Simmental cattle.

Target of Inspection	Test Methods	(Be) Worth	df	Progressive Sig. (Bilateral)	Precision Sig. (Bilateral)
CSCMM-CSCHM * Prevotella	Pearson chi-square test	73.089	2	.000	.000
	maximum likelihood ratio	81.313	2	.000	.000
	Fisher's exact test	78.878			.000
CSCMM-CSCHM * Akkermansia	Pearson chi-square test	26.146	2	.000	.000
	maximum likelihood ratio	35.750	2	.000	.000
	Fisher's exact test	29.795			.000
CSCMM-CSCHM * Blautia	Pearson chi-square test	21.600	2	.000	.000
	maximum likelihood ratio	22.324	2	.000	.000
	Fisher's exact test	21.975			.000
CSCMM-CSCHM * Bacteroides	Pearson chi-square test	11.632	2	.003	.003
	maximum likelihood ratio	11.855	2	.003	.003
	Fisher's exact test	11.624			.003
CSCMM-CSCHM * Coprococcus	Pearson chi-square test	138.042	2	.000	.000
	maximum likelihood ratio	167.730	2	.000	.000
	Fisher's exact test	161.334			.000
CSCMM-CSCHM *Faecalibacterium	Pearson chi-square test	26.860	2	.000	.000
	maximum likelihood ratio	34.395	2	.000	.000
	Fisher's exact test	30.071			.000
CSCMM-CSCHM * Morganella	Pearson chi-square test	9.726	2	.008	.005
	maximum likelihood ratio	11.133	2	.004	.005

	Fisher's exact test	9.775			.005
	Pearson chi-square test	11.659	2	.003	.001
CSCMM-CSCHM * GOUTA19	maximum likelihood ratio	16.269	2	.000	.001
	Fisher's exact test	11.907			.001
	Pearson chi-square test	10.101	2	.006	.005
CSCMM-CSCHM * Ruminococcus	maximum likelihood ratio	11.997	2	.002	.003
	Fisher's exact test	10.848			.004
	Pearson chi-square test	3.933	2	.140	.209
CSCMM-CSCHM * Nitrosopumilus	maximum likelihood ratio	5.130	2	.077	.209
	Fisher's exact test	3.473			.209
	Pearson chi-square test	58.675	2	.000	.000
CSCMM-CSCHM * [Eubacterium]	maximum likelihood ratio	67.628	2	.000	.000
	Fisher's exact test	63.588			.000
	Pearson chi-square test	76.764	2	.000	.000
CSCMM-CSCHM * [Ruminococcus]	maximum likelihood ratio	83.896	2	.000	.000
	Fisher's exact test	81.707			.000
	Pearson chi-square test	17.818	2	.000	.000
CSCMM-CSCHM * Nitrospira	maximum likelihood ratio	18.656	2	.000	.000
	Fisher's exact test	18.011			.000
	Pearson chi-square test	38.500	2	.000	.000
CSCMM-CSCHM * Klebsiella	maximum likelihood ratio	48.889	2	.000	.000
	Fisher's exact test	44.037			.000
CSCMM-CSCHM * Oscillospira	Pearson chi-square test	38.521	2	.000	.000
	maximum likelihood ratio	49.095	2	.000	.000

	Fisher's exact test	44.226			.000
	Pearson chi-square test	45.113	2	.000	.000
CSCMM-CSCHM * Dorea	maximum likelihood ratio	47.845	2	.000	.000
	Fisher's exact test	46.868			.000
	Pearson chi-square test	19.404	2	.000	.000
CSCMM-CSCHM * Dialister	maximum likelihood ratio	24.256	2	.000	.000
	Fisher's exact test	21.109			.000
	Pearson chi-square test	27.353	2	.000	.000
CSCMM-CSCHM * Cetobacterium	maximum likelihood ratio	37.341	2	.000	.000
	Fisher's exact test	31.647			.000
	Pearson chi-square test	3.346	2	.188	.197
CSCMM-CSCHM * Sutterella	maximum likelihood ratio	3.505	2	.173	.197
	Fisher's exact test	3.226			.214
	Pearson chi-square test	3.104	2	.212	.218
CSCMM-CSCHM * [Prevotella]	maximum likelihood ratio	3.237	2	.198	.218
	Fisher's exact test	3.022			.218
	Pearson chi-square test	61.308	2	.000	.000
CSCMM-CSCHM * Enterococcus	maximum likelihood ratio	80.652	2	.000	.000
	Fisher's exact test	73.410			.000
	Pearson chi-square test	8.617	2	.013	.007
CSCMM-CSCHM * Leuconostoc	maximum likelihood ratio	10.921	2	.004	.011
	Fisher's exact test	8.317			.013
CSCMM-CSCHM * Lactobacillus	Pearson chi-square test	22.610	2	.000	.000
	maximum likelihood ratio	31.061	2	.000	.000

	Fisher's exact test	25.505			.000
	Pearson chi-square test	18.081	2	.000	.000
CSCMM-CSCHM * Anaerovibrio	maximum likelihood ratio	24.996	2	.000	.000
	Fisher's exact test	19.753			.000
	Pearson chi-square test	74.083	2	.000	.000
CSCMM-CSCHM * Syntrophomonas	maximum likelihood ratio	87.068	2	.000	.000
	Fisher's exact test	84.419			.000
	Pearson chi-square test	27.267	2	.000	.000
CSCMM-CSCHM * Butyricimonas	maximum likelihood ratio	29.800	2	.000	.000
	Fisher's exact test	28.734			.000
	Pearson chi-square test	103.195	2	.000	.000
CSCMM-CSCHM * Odoribacter	maximum likelihood ratio	125.134	2	.000	.000
	Fisher's exact test	119.150			.000
	Pearson chi-square test	33.607	2	.000	.000
CSCMM-CSCHM * Citrobacter	maximum likelihood ratio	45.520	2	.000	.000
	Fisher's exact test	39.176			.000
	Pearson chi-square test	21.975	2	.000	.000
CSCMM-CSCHM * Veillonella	maximum likelihood ratio	27.355	2	.000	.000
	Fisher's exact test	23.973			.000
	Pearson chi-square test	23.775	2	.000	.000
CSCMM-CSCHM * Parabacteroides	maximum likelihood ratio	32.610	2	.000	.000
	Fisher's exact test	26.824			.000
CSCMM-CSCHM * Butyrivibrio	Pearson chi-square test	19.731	2	.000	.000
	maximum likelihood ratio	24.959	2	.000	.000

	Fisher's exact test	21.202			.000
	Pearson chi-square test	28.574	2	.000	.000
CSCMM-CSCHM * Proteiniclasticum	maximum likelihood ratio	38.947	2	.000	.000
	Fisher's exact test	33.203			.000
	Pearson chi-square test	8.125	2	.017	.015
CSCMM-CSCHM * Allobaculum	maximum likelihood ratio	10.834	2	.004	.009
	Fisher's exact test	8.385			.014
	Pearson chi-square test	22.610	2	.000	.000
CSCMM-CSCHM * Megamonas	maximum likelihood ratio	31.061	2	.000	.000
	Fisher's exact test	25.371			.000
	Pearson chi-square test	13.752	2	.001	.000
CSCMM-CSCHM * Collinsella	maximum likelihood ratio	19.131	2	.000	.000
	Fisher's exact test	14.342			.000
	Pearson chi-square test	8.603	2	.014	.007
CSCMM-CSCHM * Adlercreutzia	maximum likelihood ratio	12.061	2	.002	.007
	Fisher's exact test	8.274			.007
	Pearson chi-square test	36.622	2	.000	.000
CSCMM-CSCHM * Bulleidia	maximum likelihood ratio	43.658	2	.000	.000
	Fisher's exact test	41.736			.000
	Pearson chi-square test	13.752	2	.001	.000
CSCMM-CSCHM * Catenibacterium	maximum likelihood ratio	19.131	2	.000	.000
	Fisher's exact test	14.362			.000
CSCMM-CSCHM * Phascolarctobacter	Pearson chi-square test	24.468	2	.000	.000
	maximum likelihood ratio	31.490	2	.000	.000

	Fisher's exact test	27.301			.000
CSCMM-CSCHM * Propionicimonas	Pearson chi-square test	6.175	2	.046	.041
	maximum likelihood ratio	7.711	2	.021	.041
	Fisher's exact test	5.620			.041
CSCMM-CSCHM * Epulopiscium	Pearson chi-square test	15.892	2	.000	.000
	maximum likelihood ratio	22.039	2	.000	.000
	Fisher's exact test	17.047			.000
CSCMM-CSCHM * Lactococcus	Pearson chi-square test	34.904	2	.000	.000
	maximum likelihood ratio	47.202	2	.000	.000
	Fisher's exact test	41.028			.000
CSCMM-CSCHM * Paludibacter	Pearson chi-square test	22.229	2	.000	.000
	maximum likelihood ratio	29.117	2	.000	.000
	Fisher's exact test	25.129			.000
CSCMM-CSCHM * Lachnospira	Pearson chi-square test	24.954	2	.000	.000
	maximum likelihood ratio	34.173	2	.000	.000
	Fisher's exact test	28.490			.000
CSCMM-CSCHM * Turicibacter	Pearson chi-square test	20.319	2	.000	.000
	maximum likelihood ratio	28.002	2	.000	.000
	Fisher's exact test	22.507			.000
CSCMM-CSCHM * Steroidobacter	Pearson chi-square test	40.255	2	.000	.000
	maximum likelihood ratio	54.098	2	.000	.000
	Fisher's exact test	47.472			.000
CSCMM-CSCHM * Succinivibrio	Pearson chi-square test	16.446	2	.000	.000
	maximum likelihood ratio	21.071	2	.000	.000

	Fisher's exact test	17.483			.000
	Pearson chi-square test	13.752	2	.001	.000
CSCMM-CSCHM * Megasphaera	maximum likelihood ratio	19.131	2	.000	.000
	Fisher's exact test	14.342			.000
	Pearson chi-square test	18.081	2	.000	.000
CSCMM-CSCHM * Acetobacter	maximum likelihood ratio	24.996	2	.000	.000
	Fisher's exact test	20.071			.000
	Pearson chi-square test	24.954	2	.000	.000
CSCMM-CSCHM * Hylemonella	maximum likelihood ratio	34.173	2	.000	.000
	Fisher's exact test	28.490			.000
	Pearson chi-square test	21.458	2	.000	.000
CSCMM-CSCHM * Ruminobacter	maximum likelihood ratio	29.525	2	.000	.000
	Fisher's exact test	24.124			.000
	Pearson chi-square test	21.458	2	.000	.000
CSCMM-CSCHM * Pediococcus	maximum likelihood ratio	29.525	2	.000	.000
	Fisher's exact test	24.124			.000
	Pearson chi-square test	19.194	2	.000	.000
CSCMM-CSCHM * Brachybacterium	maximum likelihood ratio	26.493	2	.000	.000
	Fisher's exact test	21.162			.000
	Pearson chi-square test	28.574	2	.000	.000
CSCMM-CSCHM * Bifidobacterium	maximum likelihood ratio	38.947	2	.000	.000
	Fisher's exact test	32.951			.000
CSCMM-CSCHM * Gluconacetobacter	Pearson chi-square test	5.644	2	.059	.061

	maximum likelihood ratio	7.950	2	.019	.061
	Fisher's exact test	4.973			.061
	Pearson chi-square test	16.980	2	.000	.000
CSCMM-CSCHM * Bilophila	maximum likelihood ratio	23.511	2	.000	.000
	Fisher's exact test	18.336			.000
	Pearson chi-square test	21.458	2	.000	.000
CSCMM-CSCHM * Pseudaminobacter	maximum likelihood ratio	29.525	2	.000	.000
	Fisher's exact test	23.979			.000
	Pearson chi-square test	7.606	2	.022	.015
CSCMM-CSCHM * Aminobacter	maximum likelihood ratio	10.680	2	.005	.015
	Fisher's exact test	7.075			.015
	Pearson chi-square test	29.810	2	.000	.000
CSCMM-CSCHM * Brevundimonas	maximum likelihood ratio	40.567	2	.000	.000
	Fisher's exact test	34.913			.000
	Pearson chi-square test	21.458	2	.000	.000
CSCMM-CSCHM * Mucispirillum	maximum likelihood ratio	29.525	2	.000	.000
	Fisher's exact test	24.039			.000
	Pearson chi-square test	6.620	2	.037	.030
CSCMM-CSCHM * Idiomarina	maximum likelihood ratio	9.310	2	.010	.030
	Fisher's exact test	6.621			.030

Note: CSCMM is a microorganism in Chinese Simmental milk. CSCHM Chinese Simmental bovine posterior gut microbiota.

Schedule 3. Microbial chi-square test in hindgut and milk of brown cows in Xinjiang Province

target of inspection	Test Methods	(be) worth	df	Progressive Sig. (Bilateral)	Precision Sig. (bilateral)
XJBCMM-XJBCHM * Prevotella	Pearson chi-square test	97.377[a]	2	.000	.000
	maximum likelihood ratio	114.519	2	.000	.000
	Fisher's exact test	110.662			.000
XJBCMM-XJBCHM * Akkermansia	Pearson chi-square test	4.646[a]	2	.098	.108
	maximum likelihood ratio	6.204	2	.045	.108
	Fisher's exact test	4.345			.108
XJBCMM-XJBCHM * Blautia	Pearson chi-square test	85.398[a]	2	.000	.000
	maximum likelihood ratio	96.873	2	.000	.000
	Fisher's exact test	94.722			.000
XJBCMM-XJBCHM * Bacteroides	Pearson chi-square test	92.900[a]	2	.000	.000
	maximum likelihood ratio	105.890	2	.000	.000
	Fisher's exact test	102.918			.000
XJBCMM-XJBCHM * Coprococcus	Pearson chi-square test	126.549[a]	2	.000	.000
	maximum likelihood ratio	160.284	2	.000	.000
	Fisher's exact test	151.544			.000
XJBCMM-XJBCHM * Faecalibacterium	Pearson chi-square test	3.449[a]	2	.178	.190
	maximum likelihood ratio	3.619	2	.164	.179
	Fisher's exact test	3.326			.190
XJBCMM-XJBCHM * Morganella	Pearson chi-square test	2.265[a]	2	.322	.444
	maximum likelihood ratio	2.694	2	.260	.444

	Fisher's exact test	2.132			.444
XJBCMM-XJBCHM * GOUTA19	Pearson chi-square test	.646[a]	2	.724	.840
	maximum likelihood ratio	.659	2	.719	.840
	Fisher's exact test	.879			.840
XJBCMM-XJBCHM * Ruminococcus	Pearson chi-square test	18.559[a]	2	.000	.000
	maximum likelihood ratio	21.165	2	.000	.000
	Fisher's exact test	20.119			.000
XJBCMM-XJBCHM * Nitrosopumilus	Pearson chi-square test	1.985[a]	2	.371	.621
	maximum likelihood ratio	2.417	2	.299	.621
	Fisher's exact test	1.854			.621
XJBCMM-XJBCHM * [Eubacterium]	Pearson chi-square test	85.261[a]	2	.000	.000
	maximum likelihood ratio	108.683	2	.000	.000
	Fisher's exact test	100.822			.000
XJBCMM-XJBCHM * [Ruminococcus]	Pearson chi-square test	48.167[a]	2	.000	.000
	maximum likelihood ratio	54.028	2	.000	.000
	Fisher's exact test	52.152			.000
XJBCMM-XJBCHM * Nitrospira	Pearson chi-square test	17.171[a]	2	.000	.000
	maximum likelihood ratio	18.183	2	.000	.000
	Fisher's exact test	17.319			.000
XJBCMM-XJBCHM * Klebsiella	Pearson chi-square test	12.072[a]	2	.002	.001
	maximum likelihood ratio	14.964	2	.001	.001
	Fisher's exact test	12.042			.001
XJBCMM-XJBCHM * Oscillospira	Pearson chi-square test	5.202[a]	2	.074	.076
	maximum likelihood ratio	5.532	2	.063	.076

	Fisher's exact test	4.940			.082
XJBCMM-XJBCHM * Dorea	Pearson chi-square test	54.333[a]	2	.000	.000
	maximum likelihood ratio	60.898	2	.000	.000
	Fisher's exact test	58.488			.000
XJBCMM-XJBCHM * Dialister	Pearson chi-square test	3.029[a]	2	.220	.329
	maximum likelihood ratio	3.280	2	.194	.369
	Fisher's exact test	2.931			.291
XJBCMM-XJBCHM * Cetobacterium	Pearson chi-square test	19.912[a]	2	.000	.000
	maximum likelihood ratio	26.867	2	.000	.000
	Fisher's exact test	23.579			.000
XJBCMM-XJBCHM * Sutterella	Pearson chi-square test	30.368[a]	2	.000	.000
	maximum likelihood ratio	34.713	2	.000	.000
	Fisher's exact test	32.759			.000
XJBCMM-XJBCHM * [Prevotella]	Pearson chi-square test	2.180[a]	2	.336	.331
	maximum likelihood ratio	2.202	2	.333	.331
	Fisher's exact test	2.164			.331
XJBCMM-XJBCHM * Enterococcus	Pearson chi-square test	9.882[a]	2	.007	.004
	maximum likelihood ratio	12.180	2	.002	.005
	Fisher's exact test	9.575			.005
XJBCMM-XJBCHM * Leuconostoc	Pearson chi-square test	3.069[a]	2	.216	.162
	maximum likelihood ratio	3.483	2	.175	.162
	Fisher's exact test	2.965			.162
XJBCMM-XJBCHM * Lactobacillus	Pearson chi-square test	33.756[a]	2	.000	.000
	maximum likelihood ratio	45.736	2	.000	.000

	Fisher's exact test	39.805			.000
XJBCMM-XJBCHM * Anaerovibrio	Pearson chi-square test	8.746[a]	2	.013	.008
	maximum likelihood ratio	9.361	2	.009	.011
	Fisher's exact test	8.644			.008
XJBCMM-XJBCHM * Syntrophomonas	Pearson chi-square test	52.700[a]	2	.000	.000
	maximum likelihood ratio	60.056	2	.000	.000
	Fisher's exact test	57.386			.000
XJBCMM-XJBCHM * Butyricimonas	Pearson chi-square test	32.437[a]	2	.000	.000
	maximum likelihood ratio	41.075	2	.000	.000
	Fisher's exact test	37.037			.000
XJBCMM-XJBCHM * Odoribacter	Pearson chi-square test	106.927[a]	2	.000	.000
	maximum likelihood ratio	131.256	2	.000	.000
	Fisher's exact test	124.818			.000
XJBCMM-XJBCHM * Citrobacter	Pearson chi-square test	10.336[a]	2	.006	.002
	maximum likelihood ratio	14.198	2	.001	.002
	Fisher's exact test	10.248			.002
XJBCMM-XJBCHM * Veillonella	Pearson chi-square test	3.485[a]	2	.175	.166
	maximum likelihood ratio	3.735	2	.155	.166
	Fisher's exact test	3.301			.166
XJBCMM-XJBCHM * Parabacteroides	Pearson chi-square test	8.440[a]	2	.015	.012
	maximum likelihood ratio	10.348	2	.006	.009
	Fisher's exact test	8.078			.014
XJBCMM-XJBCHM * Butyrivibrio	Pearson chi-square test	6.854[a]	2	.032	.029
	maximum likelihood ratio	7.268	2	.026	.034

	Fisher's exact test	6.946			.027
XJBCMM-XJBCHM * Proteiniclasticu	Pearson chi-square test	15.127[a]	2	.001	.000
	maximum likelihood ratio	16.539	2	.000	.000
	Fisher's exact test	15.333			.000
XJBCMM-XJBCHM * Allobaculum	Pearson chi-square test	18.409[a]	2	.000	.000
	maximum likelihood ratio	21.440	2	.000	.000
	Fisher's exact test	19.978			.000
XJBCMM-XJBCHM * Megamonas	Pearson chi-square test	14.799[a]	2	.001	.000
	maximum likelihood ratio	20.206	2	.000	.000
	Fisher's exact test	15.407			.000
XJBCMM-XJBCHM * Collinsella	Pearson chi-square test	2.318[a]	2	.314	.621
	maximum likelihood ratio	3.097	2	.213	.621
	Fisher's exact test	2.024			.621
XJBCMM-XJBCHM * Adlercreutzia	Pearson chi-square test	4.318[a]	2	.115	.121
	maximum likelihood ratio	5.869	2	.053	.121
	Fisher's exact test	4.104			.121
XJBCMM-XJBCHM * Bulleia	Pearson chi-square test	6.175[a]	2	.046	.028
	maximum likelihood ratio	8.492	2	.014	.028
XJBCMM-XJBCHM * Catenibacterium	Pearson chi-square test	4.001[a]	2	.135	.182
	maximum likelihood ratio	5.546	2	.062	.182
	Fisher's exact test	3.544			.182
XJBCMM-XJBCHM * Phascolarctobact	Pearson chi-square test	37.210[a]	2	.000	.000
	maximum likelihood ratio	47.298	2	.000	.000
	Fisher's exact test	43.194			.000

XJBCMM-XJBCHM * Propionicimonas	Pearson chi-square test	.984[a]	1	.321	1.000
	maximum likelihood ratio	1.371	1	.242	1.000
	Fisher's exact test				1.000
XJBCMM-XJBCHM * Epulopiscium	Pearson chi-square test	46.297[a]	2	.000	.000
	maximum likelihood ratio	57.202	2	.000	.000
	Fisher's exact test	52.542			.000
XJBCMM-XJBCHM * Lactococcus	Pearson chi-square test	9.251[a]	2	.010	.005
	maximum likelihood ratio	12.726	2	.002	.005
	Fisher's exact test	9.183			.005
XJBCMM-XJBCHM * Paludibacter	Pearson chi-square test	26.471[a]	2	.000	.000
	maximum likelihood ratio	32.623	2	.000	.000
	Fisher's exact test	28.820			.000
XJBCMM-XJBCHM * Lachnospira	Pearson chi-square test	2.668[a]	2	.263	.446
	maximum likelihood ratio	3.453	2	.178	.354
	Fisher's exact test	2.287			.446
XJBCMM-XJBCHM * Turicibacter	Pearson chi-square test	70.103[a]	2	.000	.000
	maximum likelihood ratio	90.601	2	.000	.000
	Fisher's exact test	83.695			.000
XJBCMM-XJBCHM * Steroidobacter	Pearson chi-square test	4.941[a]	2	.085	.077
	maximum likelihood ratio	6.496	2	.039	.058
	Fisher's exact test	4.630			.095
XJBCMM-XJBCHM * Succinivibrio	Pearson chi-square test	9.251[a]	2	.010	.003
	maximum likelihood ratio	12.726	2	.002	.003
	Fisher's exact test	8.932			.003

XJBCMM-XJBCHM * Megasphaera	Pearson chi-square test	7.180[a]	2	.028	.019
	maximum likelihood ratio	9.543	2	.008	.014
	Fisher's exact test	7.187			.014
XJBCMM-XJBCHM * Acetobacter	Pearson chi-square test	1.979[a]	2	.372	.558
	maximum likelihood ratio	2.752	2	.253	.558
	Fisher's exact test	1.731			.558
XJBCMM-XJBCHM * Hylemonella	Pearson chi-square test	15.484[a]	2	.000	.000
	maximum likelihood ratio	19.371	2	.000	.000
	Fisher's exact test	16.024			.000
XJBCMM-XJBCHM * Ruminobacter	Pearson chi-square test	34.883[a]	2	.000	.000
	maximum likelihood ratio	46.493	2	.000	.000
	Fisher's exact test	40.185			.000
XJBCMM-XJBCHM * Pediococcus	Pearson chi-square test	6.067[a]	2	.048	.043
	maximum likelihood ratio	8.384	2	.015	.043
	Fisher's exact test	5.871			.043
XJBCMM-XJBCHM * Brachybacterium	Pearson chi-square test	36.272[a]	2	.000	.000
	maximum likelihood ratio	48.273	2	.000	.000
	Fisher's exact test	41.943			.000
XJBCMM-XJBCHM * Bifidobacterium	Pearson chi-square test	15.946[a]	2	.000	.000
	maximum likelihood ratio	21.740	2	.000	.000
	Fisher's exact test	16.843			.000
XJBCMM-XJBCHM * Gluconacetobacte	Pearson chi-square test	15.946[a]	2	.000	.000
	maximum likelihood ratio	21.740	2	.000	.000
	Fisher's exact test	16.806			.000

XJBCMM-XJBCHM * Bilophila	Pearson chi-square test	2.985[a]	1	.084	.246
	maximum likelihood ratio	4.143	1	.042	.246
	Fisher's exact test				.246
XJBCMM-XJBCHM * Pseudaminobacter	Pearson chi-square test	12.542[a]	2	.002	.000
	maximum likelihood ratio	17.177	2	.000	.000
	Fisher's exact test	12.715			.000
XJBCMM-XJBCHM * Aminobacter	Pearson chi-square test	7.116[a]	2	.028	.021
	maximum likelihood ratio	9.820	2	.007	.021
	Fisher's exact test	6.614			.021
XJBCMM-XJBCHM * Brevundimonas	Pearson chi-square test	5.028[a]	2	.081	.089
	maximum likelihood ratio	6.959	2	.031	.089
	Fisher's exact test	4.670			.089
XJBCMM-XJBCHM * Mucispirillum	Pearson chi-square test	8.178[a]	2	.017	.007
	maximum likelihood ratio	11.267	2	.004	.007
	Fisher's exact test	7.655			.007
XJBCMM-XJBCHM * Idiomarina	Pearson chi-square test	2.985[a]	2	.225	.497
	maximum likelihood ratio	4.143	2	.126	.497
	Fisher's exact test	2.547			.497

Note: XJBCMM is a microorganism in Xinjiang brown cow milk. XJBCHM is a posterior intestinal microbe of Xinjiang brown cattle.
